# Carbon dots as a novel photosensitizer for photodynamic therapy of cancer and bacterial infectious diseases: recent advances

**DOI:** 10.1186/s12951-024-02479-4

**Published:** 2024-04-26

**Authors:** Lingxiang Sun, Yifan Zhao, Hongyi Peng, Jian Zhou, Qingmei Zhang, Jingyu Yan, Yingyu Liu, Susu Guo, Xiuping Wu, Bing Li

**Affiliations:** 1 School and Hospital of Stomatology, Shanxi Medical University, Taiyuan, China; 2 Shanxi Province Key Laboratory of Oral Diseases Prevention and New Materials, Taiyuan, China; 3 Laboratory for Oral and General Health Integration and Translation, Beijing Tiantan Hospital, Capital Medical University, Beijing, 100069 China; 4 Taiyuan University of Science and Technology, Taiyuan, China

**Keywords:** Carbon dots, Nanotechnology, Photodynamic therapy, Antibacterial resistance, Combined treatment

## Abstract

Carbon dots (CDs) are novel carbon-based nanomaterials that have been used as photosensitizer-mediated photodynamic therapy (PDT) in recent years due to their good photosensitizing activity. Photosensitizers (PSs) are main components of PDT that can produce large amounts of reactive oxygen species (ROS) when stimulated by light source, which have the advantages of low drug resistance and high therapeutic efficiency. CDs can generate ROS efficiently under irradiation and therefore have been extensively studied in disease local phototherapy. In tumor therapy, CDs can be used as PSs or PS carriers to participate in PDT and play an extremely important role. In bacterial infectious diseases, CDs exhibit high bactericidal activity as CDs are effective in disrupting bacterial cell membranes leading to bacterial death upon photoactivation. We focus on recent advances in the therapy of cancer and bacteria with CDs, and also briefly summarize the mechanisms and requirements for PSs in PDT of cancer, bacteria and other diseases. We also discuss the role CDs play in combination therapy and the potential for future applications against other pathogens.

**Graphical Abstract:**

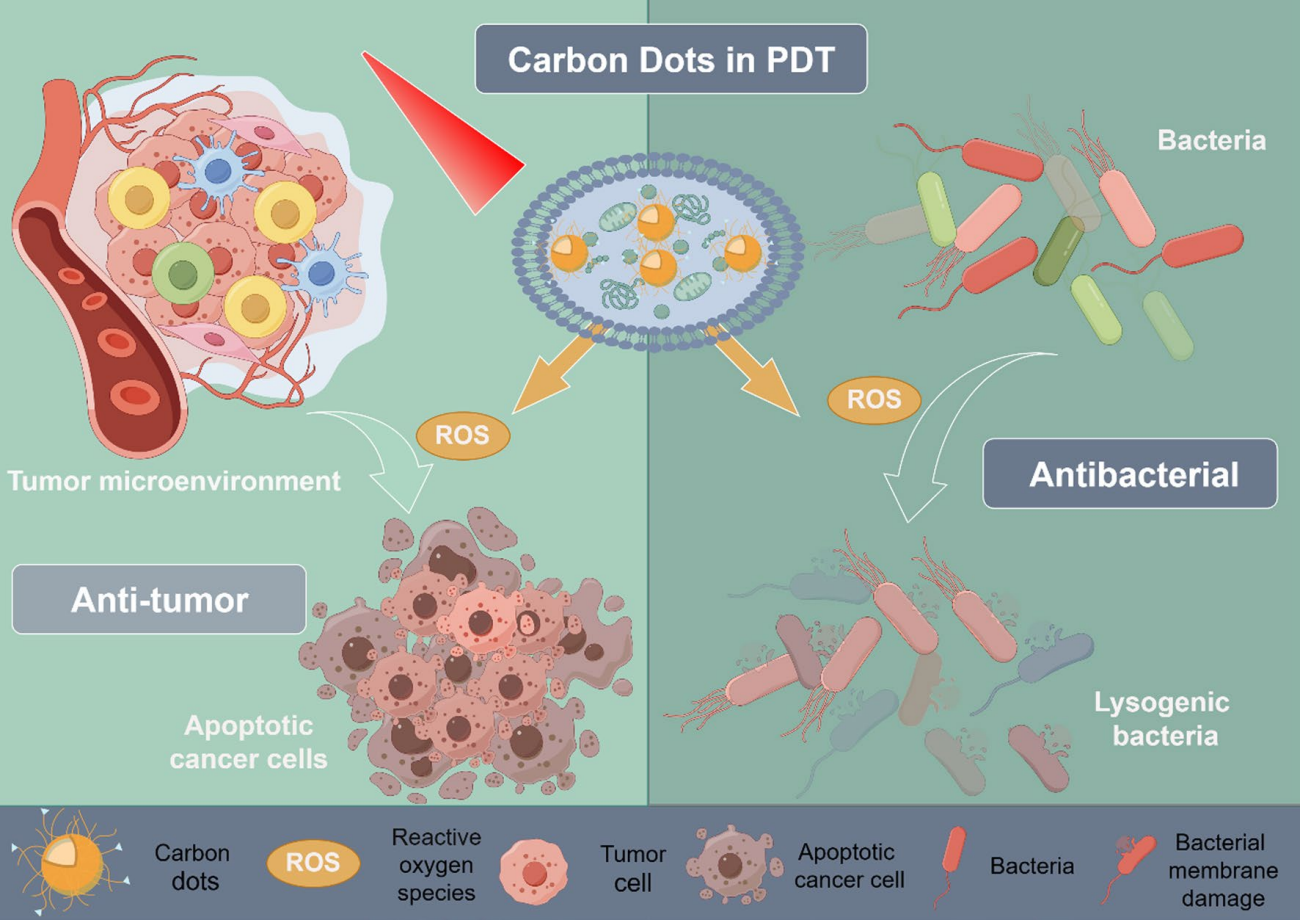

## Introduction

Malignant neoplasm is a serious threat to human life and health and its morbidity and mortality are increasing year by year [1]. The current therapy for tumors consists mainly of surgical resection, with a combination of surgery and/or radiotherapy and chemotherapy required in advanced stages [2, 3]. These treatments inevitably destroy normal tissues while killing cancer cells, causing serious side effects. As a result, the dose of chemotherapy drugs is limited and the effectiveness of the commonly used chemotherapy drugs, such as periwinkle alkaloids and paclitaxel, is greatly reduced due to the development of resistance in cancer cells [4]. At the same time, the use of high doses of chemotherapy drugs can have serious consequences for the patient’s body and mind that cannot be ignored. Bacterial infectious diseases are systemic infections caused by pathogenic or opportunistic pathogens. Antibiotics are the most commonly used means of fighting infectious diseases. Currently, the misuse of antibiotics has led to serious multi-drug resistance and the use of traditional antibiotics for many diseases is no longer sufficient for treatment, even causing delayed wound healing [5, 6]. Therefore, there is an urgent need for alternative antimicrobial methods and drugs to combat multidrug-resistant bacteria and reduce the side effects of treatment.

In the face of these challenges, photodynamic therapy (PDT), a non-invasive treatment, has attracted attention. PDT is a treatment approved by the Food and Drug Administration (FDA) and has been in development for over 30 years. It has been used clinically to treat early-stage or localized diseases and to improve quality of life and survival rates in patients with advanced diseases [7]. PDT has several potential advantages over surgery, chemotherapy and radiotherapy. PDT has shown great advantages in disease treatment: (i) PDT kills cells by generating reactive oxygen species (ROS) locally combined with its multiple sterilization mechanism, which has a short duration of action and is less likely to cause tumor and bacteria tolerance; and the timing and location of irradiation can be controlled, which reduces toxicity; (ii) PDT can kill microscopic lesions that are not visible to the naked eye at the time of surgical resection, thereby improving prognosis and preventing recurrence; (iii) PDT can be used in combination with other therapies (e.g., photothermal therapy (PTT), chemotherapy, radiotherapy, etc.) and enables fluorescence imaging, drug delivery and other applications [8–10]. However, routinely used photosensitizers (PSs) have limited their applications due to poor stability, low therapeutic efficiency and side effects on healthy cells [11, 12]. Therefore, the development of new nanomaterials for efficient PDT becomes particularly necessary.

Carbon dots (CDs) are a new type of fluorescent nanomaterial with a diameter of around 10 nm [13]. They have the advantages of simple synthesis, low toxicity, broad absorption and tunable emission, excellent photoluminescence (PL) and ease of modification, which have been extensively studied in biosensing [14, 15], bioimaging [16], disease diagnosis and therapy [17, 18]. There are two general ways to synthesize CDs: top-down and bottom-up methods. The bottom-up method allows for the preparation in large quantities from green-sourced materials, and the resulting highly biocompatible products which are more conducive to research applications in living organisms [19]. With raw materials, synthesis conditions and modification methods, it is possible to obtain CDs that meet the requirements and are thus more suitable for different fields of application [20]. CDs are capable of converting light energy into ROS or modifying PSs through their physicochemical properties thus facilitating PDT, i.e., participating in the PDT process as PSs or as carrier of PSs [21–24].

In this review, we will introduce the mechanism of PDT and the types of PSs and outline the main mechanism of killing tumor cells and bacteria. This paper mainly reviews the process of CDs participating in PDT of cancer and bacterial infectious diseases therapy, aiming to clarify the role played by CDs in mediating PDT and therapeutic efficiency.

## Therapeutic mechanisms of CDs in PDT

**Fig. 1:**
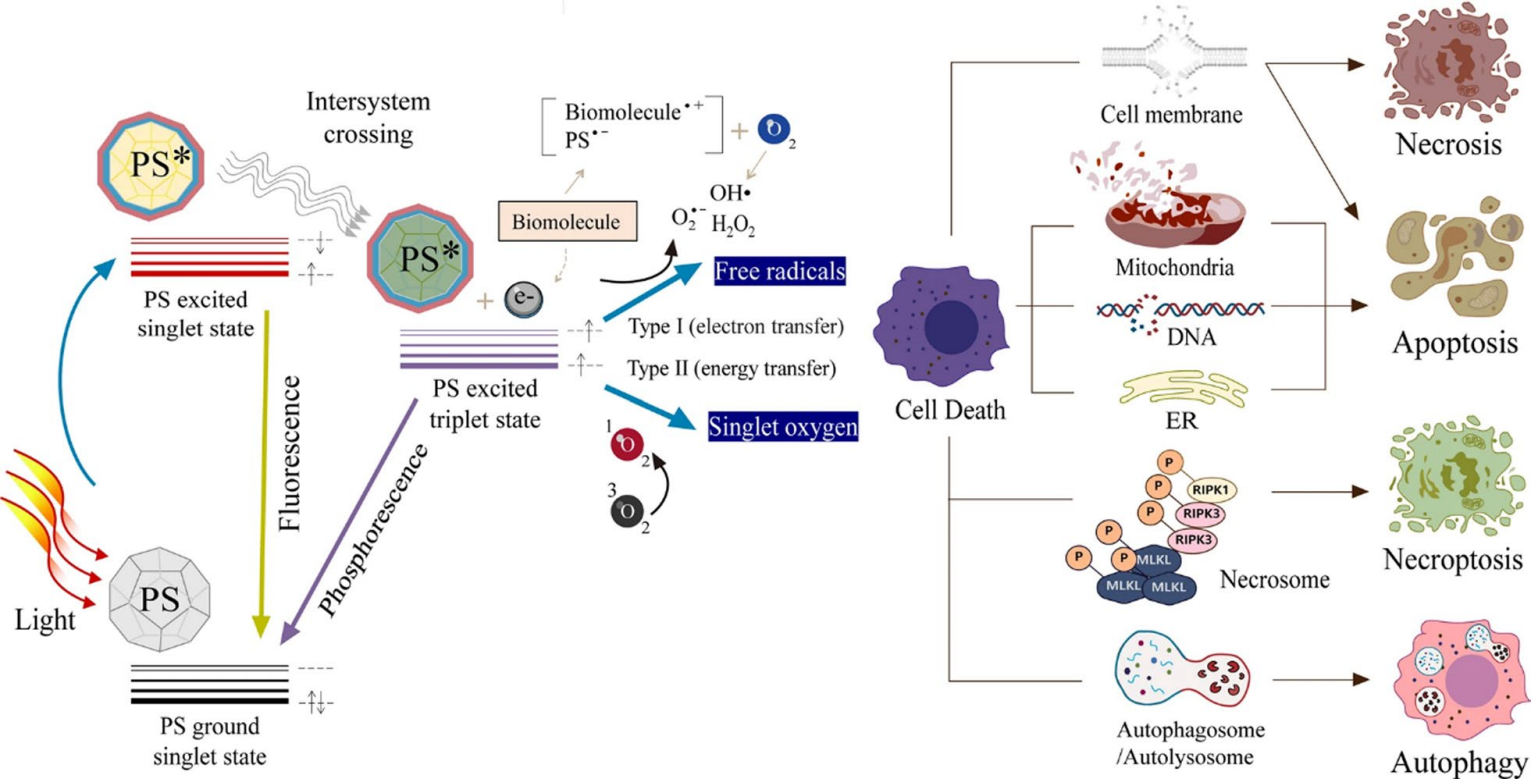
Schematic illustration of photodynamic reactions (either Type I or Type II) and cell death pathways in the process of PDT [33]. Copyright 2021, Springer Nature publishing group

The three key elements of PDT are light source, PS and O_2_. The mechanism of action is that PS absorbs energy from the ground state to the excited state when irradiated by a light source and reacts to produce ROS, which causes cytotoxicity leading to cell death [25]. The PS receives light and absorbs energy to change from the ground state to the excited state, which can result in two reactions: the first is an electron transfer between the excited state of PS and intracellular substrates (such as nucleic acids, proteins and lipids) to form free radicals, which then interact with O_2_ to produce hydrogen peroxide, superoxide anions and hydroxyl radicals (H_2_O_2_, O_2_^−^ and •OH), i.e. Type I reaction [26]. The second is the direct transfer of energy from the excited PSs to oxygen molecules through an ‘inter-system crossing’ process, resulting in the formation of singlet oxygen (^1^O_2_), i.e. a Type II reaction (Fig. 1) [27]. These two reactions can occur simultaneously and the resulting H_2_O_2_, O_2_^−^, •OH and ^1^O_2_ are collectively referred to as ROS [28–31]. The Type III reaction mechanism is that Type III PSs selectively target biomolecules, such as nucleic acids, proteins and other macromolecules, with the result that biological target molecules can be directly and effectively destroyed. Due to the stringent requirements of Type III reaction on PSs, research on the mechanism of Type III reaction is still relatively scarce [32].

**Fig. 2:**
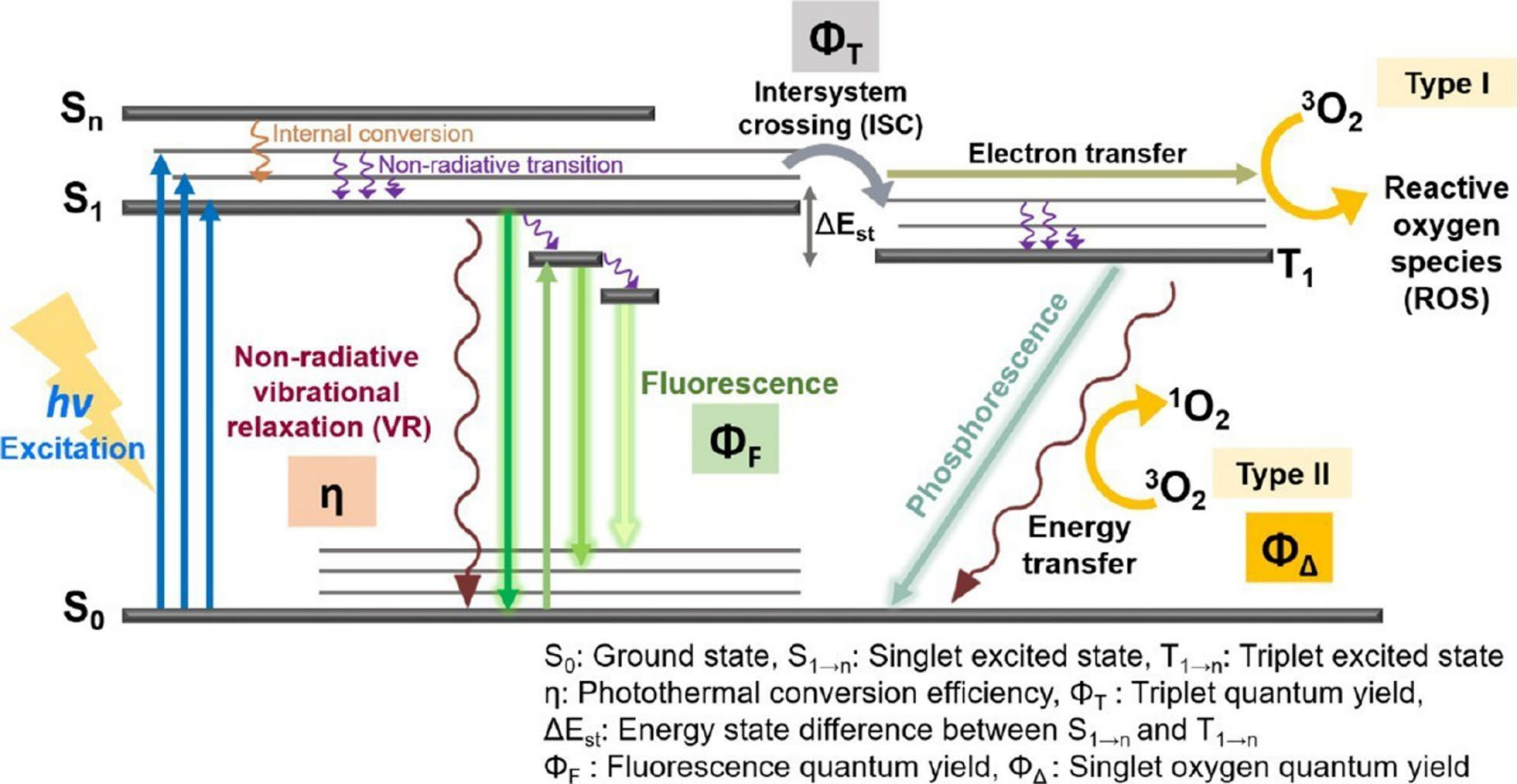
Illustrative description of mechanisms of CDs in PDT [36]. Copyright 2020, American Chemical Society publishing group

Initially, CDs are irradiated by light source and the electrons absorb energy to excite from S_0_ (ground state) to excited S_1_ (singlet excited state) and then release the energy and return to the lower energy levels through three pathways (Fig. 2): (i) non-radiative emission: in the form of heat by returning from S_1_ to S_0_, (ii) radiative emission: phosphorescence and fluorescence are the radiative processes where energy relaxation occurs in which the energy is released in the form of electromagnetic radiation light, (iii) other methods: transition from S_1_ to the excited T_1_ triplet state via intersystem crossing (ISC). The process in (iii) then involves that T_1_ can either start a free-radical chain reaction after transferring electrons to the reaction substrate with O_2_ and forming ROS such as H_2_O_2_, O_2_^−^ and •OH (Type I); or it can obtain ^1^O_2_ by transferring energy to O_2_ and extracting electrons from groups such as aromatic rings, phenolics, amines and thioethers, with the generation of the ground state by the CDs (Type II). The free radicals (H_2_O_2_, O_2_^−^, •OH and ^1^O_2_) produced by the two photochemical pathways in (iii) above are the main substances for CDs to exert the PDT effect [12, 34–36]. Little has been reported on the mechanism of CDs-mediated PDT. However, the ketonic carbonyl (–C=O), hydroxyl (–C–OH) and carboxylic (O=C–O–) groups on CDs were specifically removed, respectively, and it was found that ROS generation decreased in CDs. And the ketonic carbonyl groups decreased the most, suggesting that the presence of this oxygen-containing functional group is crucial for effective ROS generation [37]. In order to determine the relationship between the chemical structural composition and photosensitizing properties of CDs, some scholars compared top-down synthesized graphene quantum dots (GQDs) with bottom-up synthesized carbon nanodots (CNDs). The GQDs had a high PDT activity due to their aromatic core and abundant oxygen-containing functional groups. And the PDT efficacy of GQDs was significantly decreased after destroying the above structures by chemical reduction. Meanwhile, the ability of CNDs with poor photosensitizing performance was enhanced after nitrogen doping. Therefore, the chemical structural composition and surface modification of CDs, especially the structural defects in the sp^2^-carbon domain and the presence of oxygen-containing functional groups in the mechanism of ROS generation, are of great significance and instructive for the synthesis of photosensitized mediators with efficient anticancer/antibacterial properties [38].

In addition, CDs are filled with surface defects and are capable of broadly responding to the visible spectrum upon photoexcitation. Upon excitation, CDs undergo efficient charge separation and generate separated electrons and holes that can be trapped and radiatively complexed at the surface defect sites. The radiative recombination of electrons and holes leads to the formation of emissive excited states and the excited state process is responsible for the photoluminescent properties of CDs, the photodynamic effect of antimicrobials, antivirals and antitumor [39].

**Fig. 3:**
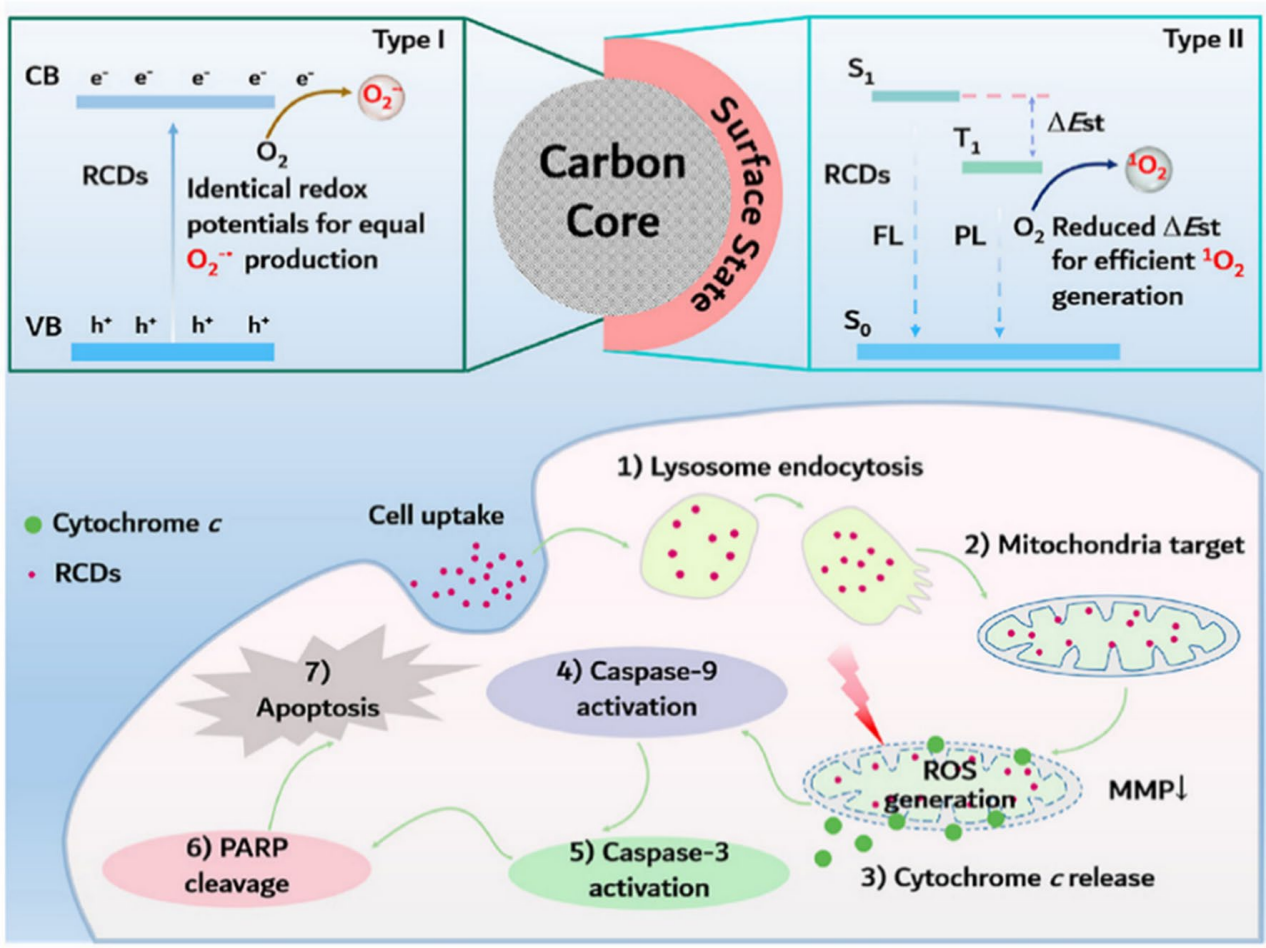
Illustration of RCDs as nano-PSs with tunable ROS generation [45]. Copyright 2022, Elsevier publishing group

It is generally accepted that the ^1^O_2_ formed by the Type II reaction is the main cause of the bio-PDT effect [40]. However, the hypoxia that may occur in larger solid tumors (pO_2_ < 5 mmHg) can diminish the anti-cancer effect of Type II PDT. Therefore, Type II PDT is more suitable for situations where O_2_ is available [41, 42]. Type I PDT, although better suited to anoxic environments, produces O_2_^−^ and H_2_O_2_ with less oxidative cytotoxicity [43, 44]. To address these issues and to achieve regulation of Type I and Type II PDT, Zhang et al. synthesized three red carbon dots (RCDs) derived from the extracts of hypericum perforatum. These three RCDs had similar core structures and different surface states, producing different ΔE_S1-T1_, while the smaller ΔE_S1-T1_ facilitated the energy transfer from S_1_ to T_1_, leading to more ^1^O_2_ production. Also, as the three RCDs had the same redox potential leading to the same O_2_^−^ production (Fig. 3). They also observed that under hypoxia, O_2_^−^ produced by the Type I PDT was significantly detected in cells, while no ^1^O_2_ was detected by the Type II PDT, demonstrating that Type II PDT was inactivated under hypoxic conditions and that Type I PDT played a dominant role in cell killing. Regarding the mechanism of apoptosis induction, they suggested the release of cytochrome *c* from damaged mitochondria-initiated cascade caspases that induce apoptotic signaling [45].

There are three main mechanisms by which PDT damages tumor tissue: (i) inducing oxidative stress causing apoptosis and necrosis of tumor cells; (ii) causing a vascular damage response; (iii) triggering an autoimmune and inflammatory response. Similarly, CDs kill bacteria by mechanisms such as (i) disrupting the cell wall/external membrane or causing mechanical damage; (ii) generating ROS-dependent and ROS-independent oxidative stress; (iii) photothermal effects; (iv) photocatalytic effects; (v) lipid-extracting effects; (vi) inhibition of bacterial metabolism; (vii) encapsulation segregation, etc. These processes are characterized by low cumulative toxicity, high spatial targeting of processes and drug resistance, independence [46]. In general, apoptosis is the main mode of cell death after PDT treatment [47]. To investigate the mechanism by which Sulphur-doped carbon dots (S-CDs) kill human tongue squamous carcinoma cells, Li et al. used Fluo-4 AM (Ca^2+^ probe) to assess the concentration of Ca^2+^ in the cytoplasm. They found that the green fluorescence was more pronounced in the S-CDs-treated group than in the 5-ALA group under laser irradiation, suggesting that the ^1^O_2_ produced by S-CDs localized in lysosomes and mitochondria-mediated the release of Ca^2+^ from internal stores into the cytoplasm and thus triggered apoptosis. In addition, they found that S-CDs induced apoptosis via the mitochondrial apoptotic pathway and Western blot results showed that the relative fluorescence intensity of the apoptosis-associated protein Bcl-2 was reduced, while the expression of Bax and caspase-3 proteins showed the opposite trend [48].

## Photodynamic therapy

### Photosensitizers

PSs are a central component of PDT and their properties primarily influence the outcome of treatment. The first kind of PSs was a water-soluble porphyrin mixture introduced by Dougherty and colleagues [49]. The main representative substances of the first generation of PSs are photofrin II and dihaemoporphyrin ethers. However, they have some inevitable disadvantages such as lack of solubility, poor tissue selectivity, long duration of photosensitivity to the skin and shallow penetration of tissue at 630 nm resulting in low therapeutic efficiency [31]. The second generation of PSs are purer in chemical composition, more stable and selective, and are optimized from hematoporphyrin, mainly including derivatives of tetrapyrroles such as porphyrins, porphyrins, endogenous porphyrin precursors and other metal phthalocyanines [50]. 5-Aminolevulinic acid (5-ALA) has been used as a formulation for surgery of clinical malignant gliomas [51]. The third generation PSs are based on the second generation modified by antibodies or nanoparticles to enhance the specificity of targeting tumor tissue and reduce systemic adverse effects, including carbon-based nanomaterials such as CDs, silicon nanomaterials, black phosphorus, micelles and vesicles as well as metal-based inorganic nanomaterials [52, 53]. The fourth generation PSs are associated with the use of porous carriers, which include mesoporous silica and metal organic frameworks (MOFs) [54–56]. Third and fourth generation PSs are that incorporate photoactive optical species such as PSs into nano or porous structures not only as functionalized carriers of PSs but also some substances that can produce ROS or modulate the conduct of PDT [57].

Among various novel PSs, carbon-based materials are as attractive as PSs due to their low cost, high stability and good biocompatibility. To obtain vaccines capable of eliciting an appropriate immune response in animals, Martin et al. used amorphous carbon nanodots (CNDs) to gently photodynamically inactivate the bacterium at wavelengths λex > 290 nm. This PS prevented damage to the outer membrane of the bacteria and elicited a stronger immune response. Due to its small particle size, CNDs were able to cross the bacterial outer membrane and respond to UV lamp irradiation by generating ROS inside the bacteria to achieve bacterial inactivation. They found that photodynamic inactivation with the addition of CNDs using UV lamps was 10 times faster than the method without CNDs and successfully verified the integrity of the bacterial membrane by agglutination and atomic force microscopy (AFM) [58].

**Fig. 4:**
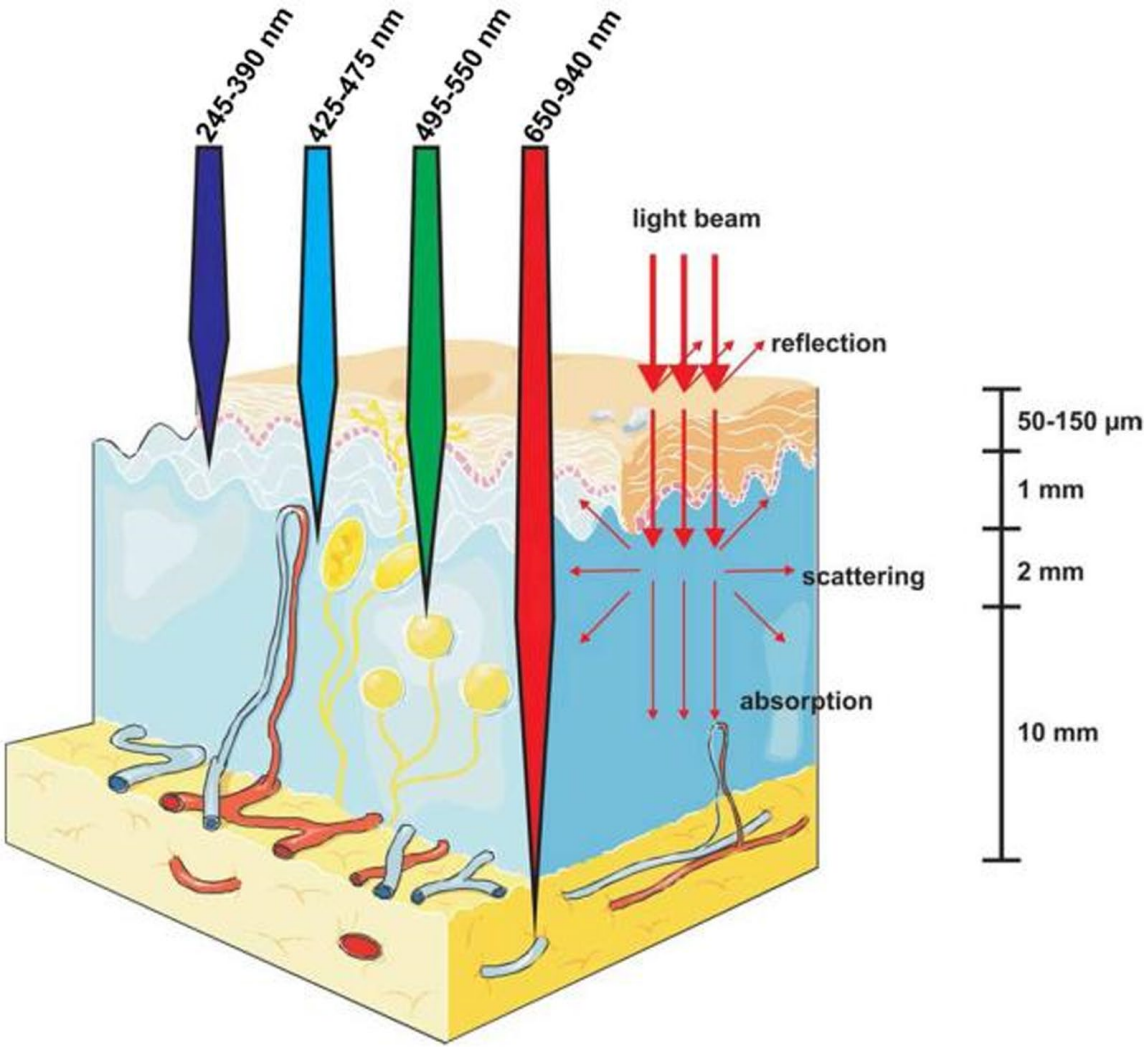
Different wavelength light penetration in biological tissues [49]. Copyright 2011, John Wiley and Sons publishing group

The intrinsic properties of PSs and the core component of PDT determine the therapeutic effect. The ideal PS should have a high light absorption coefficient, preferably in the long wavelength radiation band [59]. The longer the wavelength, the deeper the penetration in the tissue (Fig. 4). In addition, not only can PSs absorb the energy of light under irradiation, but endogenous fluorophores in living organisms such as hemoglobin and melanin also strongly absorb the visible spectrum at less than 650 nm to produce self-luminescence. Optimal excitation wavelengths of PS for PDT applications are preferably greater than 650 nm. However, at wavelengths greater than 900 nm, the scattering and absorption of light by water molecules in the tissue increases. Therefore, to obtain the optimum depth of tissue penetration, the optimal absorption spectrum of the PS should be in the wavelength range of 650–900 nm. This range of tissue penetration depths is called the “optical window” or “histological window” [60]. To protect normal tissues, PSs should have low dark toxicity and minimal effect on tissues under non-light conditions [54].

The good optical properties and high quantum yield (QY) of CDs lay the foundation for their applications as highly efficient PSs. In general, CDs prepared from different raw materials or synthesis methods have different optical absorption behaviors [61]. However, CDs usually have a wider range of absorption, showing strong absorption phenomena in the UV region (200–400 nm). This absorption is related to the π–π* leaps of the C=C bond and the n–π* leaps of the C=O bond, and is also affected by the synthesis methods, raw materials and surface functional groups [62, 63]. PL, the ability to emit photons due to photon absorption and subsequent excitation of electrons at higher energy levels, is the most attractive feature of CDs and is closely related to their structural composition. Typically, CDs consist of a carbon core and surface domains, both of which are responsible for different fluorescence properties [64]. Specific PL mechanisms may be quantum confinement effect (QCE), quantum size effect (QSE), surface states, crosslink-enhanced emission effect (CEE), and molecular states, but none of them have been precisely determined yet [65–69]. In addition, the PL of CDs is also affected by extrinsic factors such as the use of single or multiphoton stimulation, as well as changes in the solvent, pH and the concentration itself [70].

CDs undergo photoexcitation after receiving light energy and induce a series of events such as radiative relaxation and photodynamic process. Non-radiative relaxation, radiative leaps and singlet to triplet state leaps through higher photon absorption correspond to the production of heat, fluorescence and ^1^O_2_, respectively [36, 71, 72]. Each of these phenomena occurs by dissipating excess photon energy within the photoexcited CDs, i.e., acting as an energy donor. In general, to balance the energy dispersion, PSs with higher ^1^O_2_ yields (Φ_Δ_) correspond to lower fluorescence QY (Φ_F_), as shown in Fig. 2 [36]. QY is the number of emitted photons relative to the number of absorbed photons and indicates the ability of CDs to emit fluorescence. The QY of CDs is largely dependent on the synthesis process and the chemical modifications on the surface, which can greatly affect the high contrast of the CDs for bioimaging [73]. Afterglow imaging CDs for imaging-guided PDTs are already available, enabling tumor suppression based on non-fluorescence-dependent imaging and solving the energy balance problem described above. CDs were synthesized via chlorin e6 (Ce6) and polyethylene imine (PEI). The obtained CDs emit intrinsic near-infrared (NIR) light with a lifetime of more than 2 h at 680 nm, which could provide tissue penetration of about 10 mm. Meanwhile, the results of in vivo PDT treatment showed that tumor growth in the treatment group was significantly inhibited by CDs + laser and induced to produce severe cell necrosis [74].

Current studies have shown that heteroatom doping is a common means of modulating the luminescence of CDs, and the QY of CDs can be significantly enhanced by doping with nitrogen (N) [75], while CDs with wide bandgaps can be synthesized by doping with sulfur (S) [76]. Furthermore, phosphorus (P)-doped CDs show green fluorescence with enhanced imaging signal and low background interference [77, 78]. Researchers have altered the absorption and emission peak positions of CDs by doping elements to improve the fluorescence properties and photosensitivity of the materials. Such CDs can act as PSs in direct response to irradiation from NIR light sources, generating ROS through an electron or energy transfer process [79]. Kuo et al. then prepared GQDs containing higher N bonding components to comparing the effect of N content on the therapeutic effect of PDT. The results showed that GQDs with high N content produced more ROS under the same irradiation conditions (670 nm, 0.1 W/cm^2^) and exhibited close to 100% killing of E. coli compared to N-free and N-poor GQDs. The mechanism of action may be that the higher N in the CDs improves the photosensitivity and energy absorption in the NIR region and acts through the synergistic production of ROS and RNO (reactive nitrogen) [80]. Therefore, the CDs used for PDT are required to have a greater absorption of light in NIR region, since NIR light sources are more efficient in penetrating biological tissues and treating.

As a kind of inorganic PSs, CDs are able to produce a wider variety of ROS types due to the structure and easy electron transfer characteristic [81]. At the same time, CDs have the advantage of broadband absorption and can respond broadly to the stimulation of light sources in different wavelengths, which increases the ROS yield with little limitation by the excitation wavelength [80, 82]. CDs are easy to modify and functionalize, such as atom doping (e.g., N, P, S, F and P) and functional group (e.g., carbonyl, carboxyl, hydroxyl, and amine groups) modifications, and can be synthesized to control their optical activity (PL, QY, energy level, spectral range and photosensitivity, etc.) as well as to obtain good aqueous solubility [83]. As PSs, CDs overcome the limitations of poor water solubility, toxicity, poor stability as well as low photosensitizing activity of traditional PSs, while their own good optical properties can realize imaging-guided PDT to some extent [36].

### Light source

The light source used for PDT should primarily consider the type of light required, the desired depth of tissue penetration and the absorption spectrum of PS, which may determine the effectiveness of PDT. In addition, the energy density, power density and duration of illumination of the light source are also important determinants and the relationship between the three is as follows: duration of illumination (s) = energy density (J/cm^2^) / power density (W/cm^2^). Most of the PSs currently used in clinical practice are light emitting diodes (LED), lasers and semiconductor lasers. In general, the PDT effect achieved by excitation with narrow-spectrum light sources is stronger, all other conditions being equal in control. For isolated, small lesions the laser is more effective as a light source, for multiple, large lesions the LED light with a large spot is more suitable [84, 85]. Lasers have been used in a wide variety of PDT applications [86]. The main advantages of laser systems are the ability to radiate monochromatic light and the ability to control the depth of penetration through energy regulation [87]. Of several types, laser diode is currently the preferred choice because of its small size, long life and high efficiency of laser utilization, as well as the adjustable optical power output. However, PDT laser systems have complex electronic circuitry and are very expensive. And due to their narrowband devices can only emit monochromatic behavior at characteristic wavelengths, the applications for different PSs are greatly limited [88, 89]. Broad-spectrum LED light sources have emerged as an important viable solution for reducing the cost of laser PDT and adapting CDs for applications with broad-spectrum absorption. LED-mediated PDT applications have also shown promising results in the treatment of different types of diseases [90, 91]. LED light sources can be fixed to the desired wavelength by temperature and current control methods, offering the advantages of low cost and good optical stability [92].

**Fig. 5:**
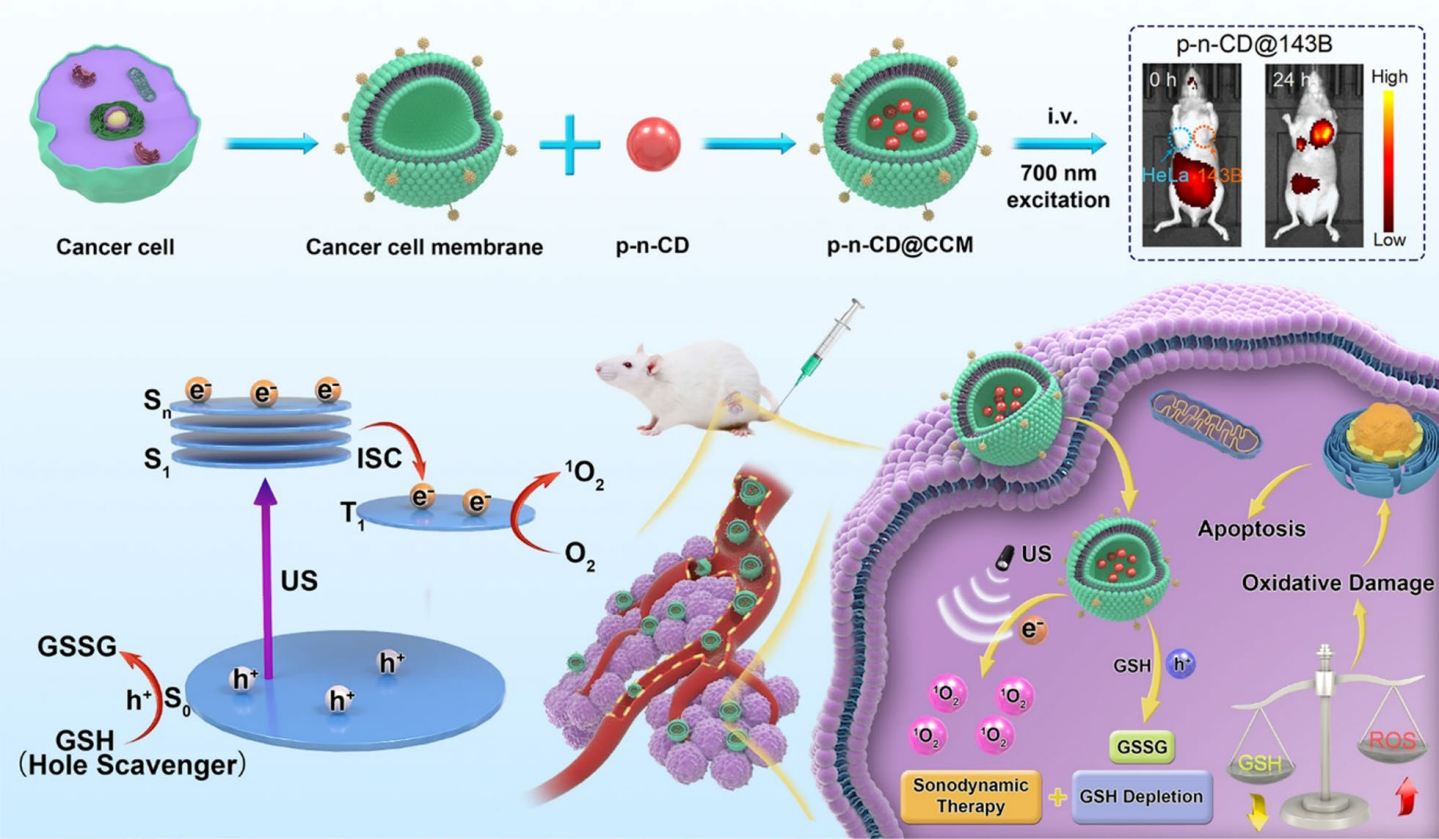
NIR phosphorescent CDs for tumor-specific NIR imaging and precise sonodynamic therapy [97]. Copyright 2022, Springer Nature publishing group

X-rays have already been investigated as a “light source” for PDT, as they carry high energy and penetrate deep into the tissue, promising the diagnosis and treatment of deep tumors. X-ray doses for X-ray induced photodynamic therapy (X-PDT) are typically 0.18–10 Gy [93]. X-ray and internal light-excited PDT have no limitations in terms of penetrating tissue but suffer from low energy transfer efficiency and insufficient generation of ^1^O_2_ [60]. In addition to using X-rays, low-intensity ultrasound energy can also be used as a “light source” to locally activate sensitizers due to its better penetration, a treatment modality also known as sonodynamic therapy (SDT) [94]. The ultrasound used is usually between 1 and 2 MHz with a power density of 0.5 to 10 W/cm^2^. The ultrasound-activated cavitation effect produces ROS-induced apoptosis, necrosis and autophagy [95]. Apart from this, some chemotherapeutic agents are enhanced by ultrasound, which allows for the combination of multiple therapeutic modalities [96]. NIR-emitting CDs can be used as phosphorescent sonosensitizers to build NIR imaging-guided sonodynamic therapy platforms as well as to enhance charge separation kinetics. CDs with p–n junction have bright long-lived (11.4 μs) phosphorescence, enabling precise tumor treatment using cancer cell membranes as targeting agents and delivery systems (Fig. 5) [97].

## Applications

Previous studies have shown that carbon-based nanomaterials are highly efficient PSs for the production of ROS under light-activated photodynamic processes for cancer and bacterial infectious diseases. CDs, as excellent PSs or PS carriers because of their good water solubility, low toxicity and high singlet oxygen quantum yields. When used as a carrier, CDs can improve the lack of water solubility of PSs, improve biocompatibility and alter diffusion mechanisms to enhance the uptake of PSs by cells. In addition, the enhanced permeability and retention (EPR) properties of nano-scale CDs can target organizations, leading to targeted PDT effects and thus limiting phototoxicity to normal tissues [57, 98].

### In tumor therapy

CDs have a π–π conjugated structure with a core consisting of graphitized sp^2^ hybridized carbon and a shell rich in organic functional groups such as carboxyl, amino and hydroxyl groups, which makes CDs easy to undergo a variety of surface modifications. CDs have great potential for direct use as PS-mediated PDT.

At present, the main developments of CDs for anti-cancer PDT are:(i) as nano PSs [99]; (ii) as PS carriers of PDT [100, 101]; (iii) combined with PTT and chemotherapy [45, 102].

#### CDs as photosensitizers and carriers

The use of nanoscale CDs as PSs and PS carriers aims to address many of the current problems with PSs, such as deficiencies in solubility, stability and bioavailability. As PSs, CDs can maximize the generation of sufficient ROS in response to light and have higher photosensitizing activity. As carriers, CDs have a high surface area to volume ratio and are capable of loading large amounts of PSs. At the same time, the properties of CDs stability and good solubility can be transferred to PS to improve its bioavailability.

Mg-free chlorophyll is a biomass similar to chlorophyll with strong NIR absorption and emission. However, it has a porphyrin structure that is hydrophobic and cannot be directly applied to PDT [103]. CDs with NIR luminescence were obtained by microwave method using Mg-free chlorophyll as a precursor by Wen et al. They used DSPE-mPEG2000 to increase hydrophilicity through hydrophobic-hydrophobic interactions. The maximum emission peak was at 680 nm, indicating that CDs could achieve NIR bioimaging while having a high singlet oxygen quantum yield (0.62). Significant tumor volume reduction and histological pathological changes were observed in the PDT group, demonstrating that green-derived CDs can be used as low-toxicity and highly effective PSs for cancer treatment [104]. Similarly, to improve the water solubility of riboflavin as a natural PS, riboflavin-derived CDs were prepared. Such CDs not only retained the optical properties of riboflavin as PSs but also improved the water solubility and fluorescence quantum yield. In PDT, the viability of 4T1 cells decreased to 31% after 20 min using laser irradiation (365 nm, 60 mW/cm^2^), whereas the viability of the group without CDs decreased only 13% after irradiation. This suggests that natural biological to can be adequately used for PDT after being treated to make CDs [105].

To achieve active targeting of PSs to tumor cells, Zhang et al. used hyaluronic acid (HA), which is capable of targeting CD44, as the carbon source for hydrothermal methods of carbon dots (HA-CDs). In addition, HA-CDs exhibited highly selective fluorescence imaging and precisely selective killing upon laser irradiation of A549, B16 and 4T1 cells highly expressing CD44 [106]. In addition to actively targeting receptors on the surface of the cell membrane, the cell nucleus also becomes a potential target. Using citric acid and tea polyphenols with various anti-cancer properties as raw materials, Yang et al. synthesized water-soluble CDs with red emission. CDs could generate hydroxyl radicals (•OH) under 400–500 nm LED light (15 mW /cm^2^) and cause a significant reduction in tumor cell activity and size with Type I reaction. Interestingly, CDs could enter the nucleus and localize in the nucleolus region, promising a potential red fluorescent probe [107]. Pang et al. synthesized CDs capable of localizing to the nucleolus for more efficient photocytotoxicity. They found that these CDs were able to target RNA in the nucleus of 4T1 cells and generate large amounts of ROS in the nucleolus that subsequently diffused into the cytoplasm and that this targeting could be maintained for nearly 5 h. At the same time, the 2 nm diameter of the CDs ensures rapid clearance by the kidney, which had a favorable biosafety and application potential [108].

The CDs synthesized by He et al. from diketopyrrole (DPP) and chitosan (CTS) retained the ability of DPP to generate ^1^O_2_ and improved the water solubility. DPP is a stable, conjugated structural dye with good fluorescence quantum yield and its derivatives have been widely used in PDT [109]. In this study, the quantum yield of ^1^O_2_ from DPP CDs was 27.6%, which was reflected in a solid tumor model of anti-H22 (hepatocellular carcinoma) cells, with a significant reduction in tumor size in the DPP CDs + 540 nm laser irradiation group. Meanwhile, the results of the Calcein-AM/PI assay and the 3-(4,5-di-methylthiazol-2-yl)-2,5-diphenyltetrazolium bromide (MTT) viability assay were consistent, confirming that 50% of cells were inhibited by irradiation at the same concentration while the survival rate of non-irradiated cells was above 90% [110].

As a carrier of PSs, the addition of CDs makes the whole system stable and efficient. Ce6 is a PS with a high singlet oxygen quantum yield (0.75), but it has poor water solubility and a short half-life in blood [111]. Songeun Beack et al. chemically coupled CDs, HA and Ce6 to solve these problems [112]. In this study, as HA is a good transdermal delivery vehicle, the Cdot-Ce6-HA complex penetrated the cancerous skin tissue via the overexpressed HA receptor CD44 on the surface of cancerous skin cells [113]. In addition to its excellent targeting properties, the Cdot-Ce6-HA complex was significantly more water soluble and effective in treating cancer compared to pure Ce6. Again, coupling CDs to Ce6, Huang et al. exploited the fluorescence resonance energy transfer (FRET) mechanism between the two to enhance PDT and photosensitizer fluorescence detection. It could be observed that the fluorescence emission of CDs in the 450–650 nm range overlaps with the absorption of Ce6 in the same range, meaning that the fluorescence emission of CDs could indirectly excite Ce6 to enhance the fluorescence intensity (35-fold). With the presence of CDs, the efficiency of Ce6 production of ^1^O_2_ was increased and bright fluorescence could be detected in mice, especially in the tumor region, enabling a combination of fluorescence diagnosis and therapy [100].

Many deep tumor tissues cannot receive effective PDT due to the limitation of penetration depth. To achieve effective treatment depth, the use of NIR light to activate PSs is an encouraging approach. Nilanjon Naskar et al. proposed the excitation of carbon dots-coupled human serum albumin-Ru complexes (HSA-Ru-Cdot) with low energy NIR two-photon to achieve efficient ^1^O_2_ generation at 810 nm. In this study, CDs were used for two-photon absorption in NIR region. The CDs’ fluorescence lifetime imaging microscopy (FLIM) images showed a double exponential fluorescence decay, which implied a shortened fluorescence lifetime of CDs, demonstrating the presence of efficient FRET in HSA-Ru-Cdot. HSA-Ru-Cdot had mitochondrial subcellular targeting capabilities and was taken up by cancer cells via intranuclear body release. Cell viability of A549, HeLa was reduced by 22%-55% at 15 min of 810 nm LED light irradiation (7 mW/cm^2^) and these results provided a promising direction for PDT [114].

**Fig. 6:**
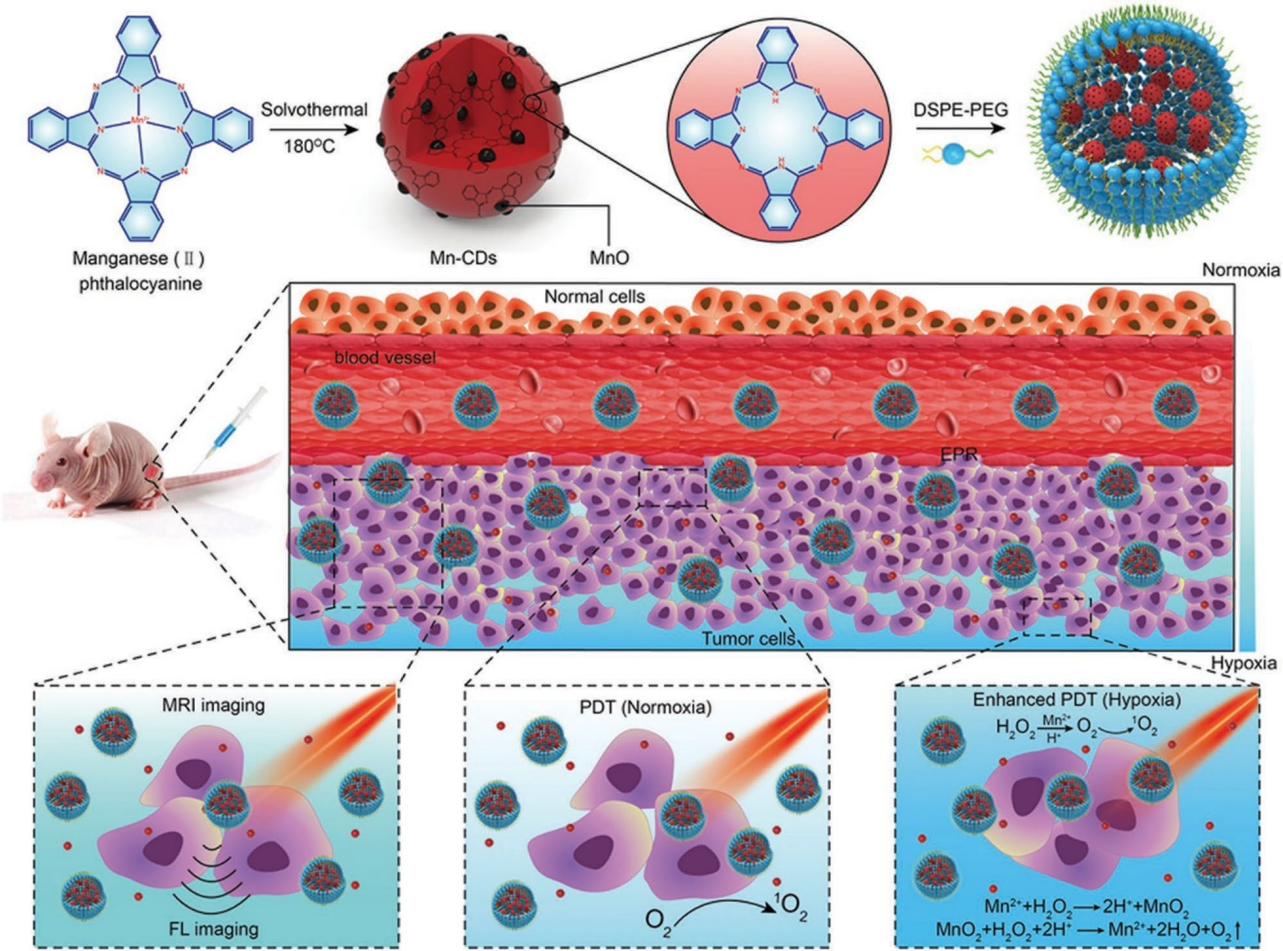
Mn-CD assembly as an acidic H_2_O_2_-driven Oxygenerator to enhance the anticancer efficiency of PDT in a solid tumor [117]. Copyright 2018, John Wiley and Sons publishing group

The PDT process is oxygen-dependent, but the lack of oxygen in the tumor microenvironment limits the therapeutic efficacy of PDT [115, 116]. To address this issue, Jia et al. developed magneto-fluorescent carbon dots (Mn-CDs) that could efficiently catalyze the generation of oxygen from H_2_O_2_ while generating ^1^O_2_ (quantum yield of 0.40), and this Mn-CDs could also guide PDT in tumor-bearing mice while enabling dual-peak MR/fluoroscopic imaging. In this study, Mn-CDs from manganese phthalocyanine (Mn-Pc) were co-assembled with DSPE-PEG to enhance their water solubility and biocompatibility. Emission spectra of Mn-CDs showed an emission peak at 745 nm under 690 nm excitation, demonstrating the NIR emission capability of Mn-CDs. More interestingly, Mn-CDs can catalyze the production of oxygen from acidic H_2_O_2_, overcoming the impact of hypoxic tumor microenvironment on PDT (Fig. 6). Also, free Mn^2+^ can be used as a developer for nuclear magnetic imaging and the measured relaxivity value of the Mn-CDs complex is approximately 6.97 mM^−1^ s^−1^, similar to other Mn^2+^-containing developers [117].

**Fig. 7:**
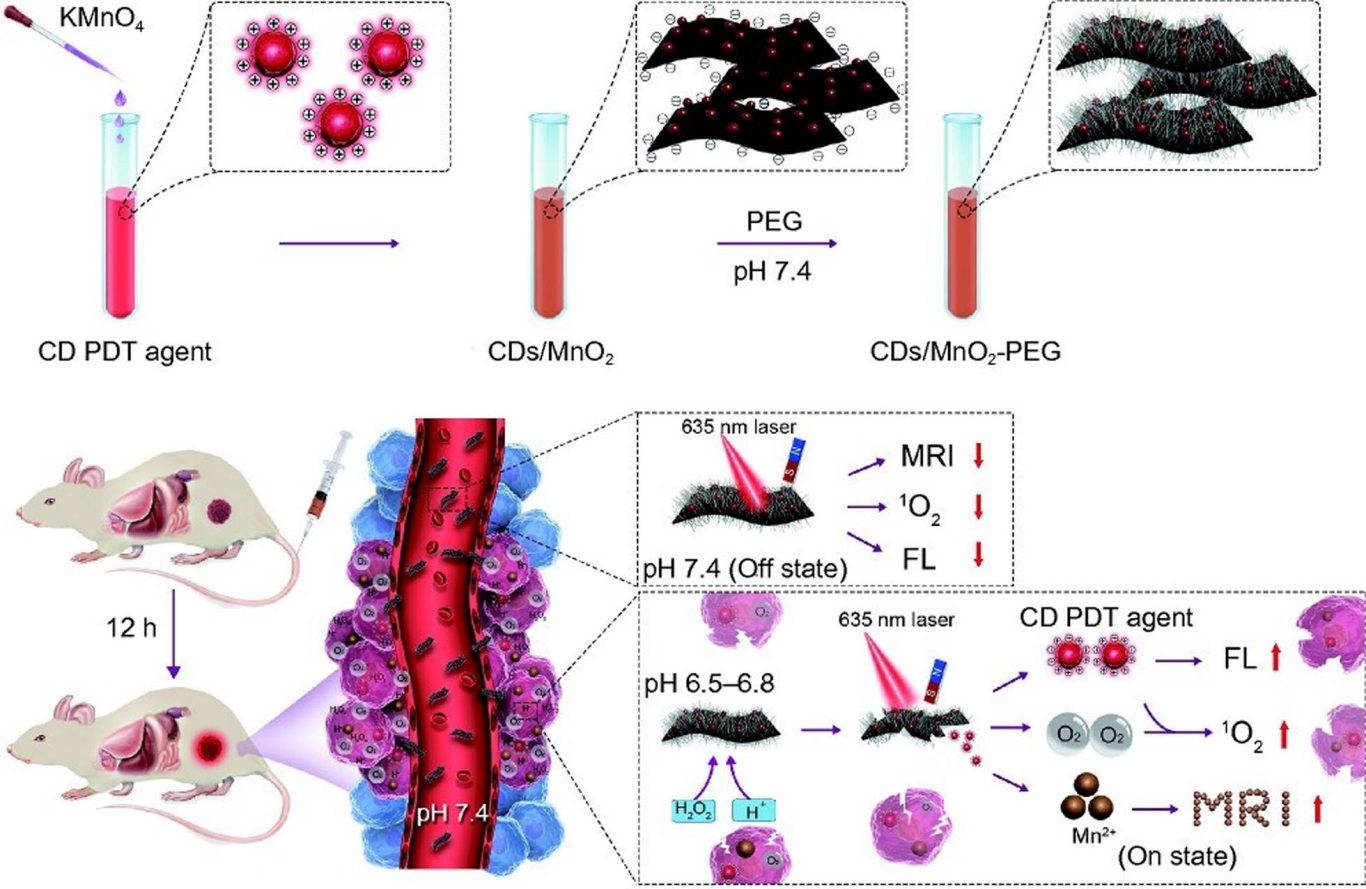
CDs/MnO_2_-PEG nanohybrids as a multimodal theranostics for the MR/FL imaging-guided PDT [118]. Copyright 2018, Springer Nature publishing group

Under physiological conditions (pH 7.4), the polyethylene glycol-modified CDs/MnO_2_ exhibited burst fluorescence, weak ^1^O_2_ generation and low MRI signal. When acidic H_2_O_2_ was introduced into the tumor microenvironment, it could enhance the fluorescence of CDs by decomposing MnO_2_ and generate large amounts of O_2_ in situ for PDT and the generated Mn^2+^ could also be used as MRI contrast agent for MRI/FI multimodality imaging (Fig. 7**)**. CDs/MnO_2_-PEG nanohybrids have achieved a singlet oxygen quantum yield of more than 1.3 due to solving the problem of hypoxia in solid tumors, while maintaining the “off” state in the physiological environment to achieve the targeting effect on the acidic environment of tumors [118].

#### Combination therapy with PDT

Phototherapeutics, a technique combining photoinduced diagnostics and therapeutics, has great potential for multimodal cancer imaging and effective treatment. Limited by the therapeutic depth of PDT, even the preparation of NIR emitting CDs cannot meet the demand, so it is necessary to combine PDT with other therapies to fill the gap.

Despite the excellent PDT effect of CDs, there are still some drawbacks including low drug enrichment efficiency and low permeability. It is important to maintain the enhanced therapeutic effect without increasing the amount of CDs. Similar to PDT, PTT is another noninvasive therapeutic strategy. PTT converts photon energy into thermal energy and uses the heat to kill cancer cells. Combining PDT with PTT can achieve better treatment results.

**Fig. 8:**
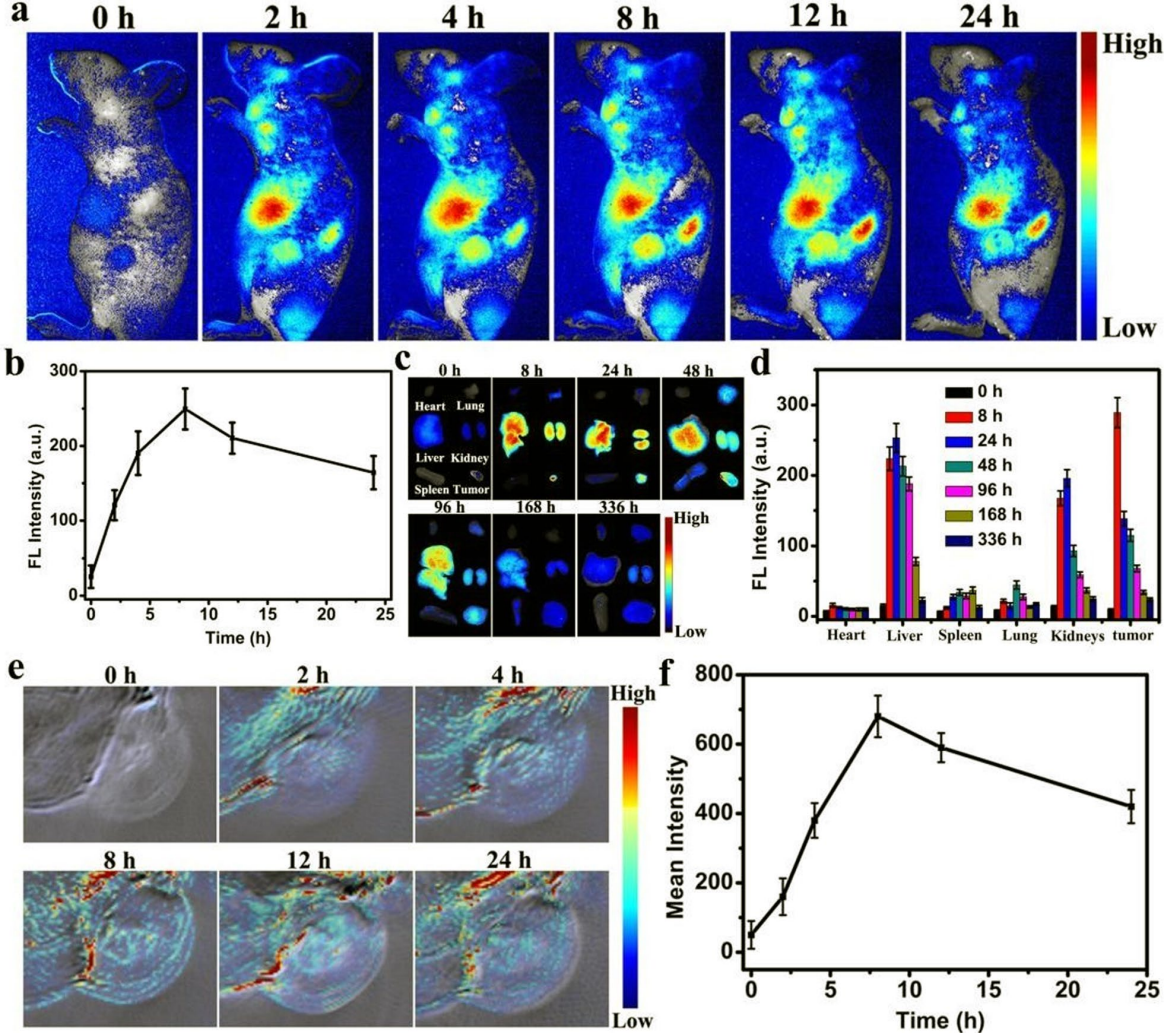
Fluorescence and photoacoustic imaging signals in vivo*.* **a** In vivo FL imaging of mice post-i.v. injection of HBCDs in PBS. **b** FL intensities of tumors in (**a**). **c** Ex vivo FL imaging of tumor and major organs at different time points post-i.v. injection of HBCDs in PBS. **d** FL intensities of major organs and tumor in (**c**). **e** In vivo PA imaging of mice post-i.v. injection of HBCDs in PBS. **f** PA intensities of tumors in (**e**). Data are expressed as means ± s.d. [120]. Copyright 2018, Elsevier publishing group

Using 1,3,6-trinitropyrene and Na_2_SO_3_ as precursors, Lan and co-workers synthesized CDs with a fluorescence quantum yield of 49% by hydrothermal methods. CDs could produce ^1^O_2_ (with a quantum yield of 7.2%) and thermal energy under 800 nm laser irradiation with a photothermal conversion efficiency of 52.3%. After intravenous injection of CDs, an increase in the intensity of the photoacoustic signal in the tumor area was observed over time and reached a maximum of 6 h after injection. In animal studies, the temperature at the center of the tumor laser spot in the CDs + irradiated group could reach 65 °C after 10 min, while the tumor volume was significantly reduced [119]. Similarly, CDs synthesized by Jia et al. using Hypocrella Bambusae (HB) as a precursor also had a dual guiding role for fluorescence and photoacoustic imaging in PDT/PTT. This green-sourced CDs had the advantage of being low-cost, red-light emission (maximum peak at 610 nm) and biocompatible, while Hypocrellins extracted from HB had also been shown to have photodynamic antiviral and anticancer activity. The temperature of the aqueous solution of CDs rose by 26.9 °C after 10 min of 635 nm laser irradiation at 0.8 W/cm^2^ and the singlet oxygen quantum yield of CDs was 0.38. After intravenous injection in tumor-bearing mice, clear fluorescence imaging and photoacoustic image signals were collected from the tumor sites of mice due to the enhanced EPR effect, while the tumor site temperature rose to 56.4 °C (Fig. 8) [120].

To exploit the role of multimodal imaging in guiding PDT and PTT, Jia et al. developed a multifunctional gold nanorod @silica–carbon dots (GNR@SiO_2_-CDs) phototherapeutic agent to guide intravenous drug delivery through high sensitivity and good spatial resolution of fluorescence/photoacoustic imaging. GNR@SiO_2_-CDs generated ^1^O_2_ under 635 nm laser irradiation (0.1 W/cm^2^), which causes ^1^O_2_ of the standard fluorescent indicator 2’,7’-Dichlorodihydrofluorescein diacetate (DCFH-DA) to emit bright green fluorescence upon oxidation. In contrast, GNR@SiO_2_-CDs generated heat to raise aqueous solutions from 7.3 to 22.6 °C under 808 nm laser irradiation (0.5 W/cm^2^), indicating their potential for synergistic PDT/PTT of cancer at low laser power doses. Photoacoustic images of GNR@SiO_2_-CDs in vivo clearly show tumor microstructures with high contrast, while as a fluorescent developer, GNR@SiO_2_-CDs also performed quite sensitively [121]. Hemin can be used as an anti-cancer drug to cause apoptosis and as a PS to absorb 220–500 nm UV–VIS light to kill cancer cells. Coupling with CDs enhanced the FRET effect on hemin and solved the problem that hemin could not penetrate deep tissue to absorb NIR light. At 10 min of 650 nm laser irradiation, the water temperature increased by up to 27 °C and the resulting ROS caused a significant decrease in the intensity of the DPBF characteristic peak at 410 nm [122].

PTT does not require the use of O_2_ in tissues to produce cytotoxicity, so combined PTT can alleviate the oxygen dependence of PDT. However, PTT and PDT can be cumbersome in practice because they require the use of light sources of different wavelengths and intensities. Also, local laser irradiation determines that both treatment modalities are ineffective for metastatic tumors.

Combining PDT with chemotherapy (CHT) can achieve a better tumor treatment effect. The more commonly used chemotherapy drug in combination is currently doxorubicin (DOX). Li et al. used CDs as nanocarriers to load the anticancer chemotherapeutic drug DOX after combining it with 5-ALA and the encapsulation rate of DOX reached 83.0% [123]. CDs had two tasks in this system: as a nanoplatform for drug loading and as a fluorescent probe for diagnosing cancer cells. Fourier transform infrared (FTIR) spectroscopy showed that CDs were bound to 5-ALA and DOX via an EDC/NHS coupling reaction and hydrogen bonding, respectively. In acidic media, the carrier swelled and broke the linkage with 5-ALA and DOX was rapidly protonated and released, a property that facilitated drug release in the acidic tumor microenvironment. They observed that the synergistic effect of laser combined with chemotherapeutic drugs increased mortality in breast cancer MCF-7 cells, and a large number of studies had also reported that chemotherapeutic drugs and PSs intercalate through DNA and disrupt topoisomerase-mediated DNA repair [124]. Similarly, the large number of functional groups on the surface of CDs, which can generate ROS and ^1^O_2_ under light, and the anthracycline and amino groups of DOX enable the combination of PDT and chemotherapy through π–π superposition and electrostatic interactions, respectively. Interestingly, they found that the Zeta potential of CDs/DOX depended on the Zeta potential of DOX and the electrostatic interactions between CDs and DOX, while the products of high ethylenediamine (EDA) ratios led to an increase in overall cell membrane permeability due to high amine doping. Their results indicated that CDs/DOX (CA: EDA = 1:2) exhibited the strongest cytotoxicity in HeLa cells compared to CDs, free DOX [102].

PDT used in combination with chemotherapeutic agents is increasingly demanding for targeting, and the EPR effect of passive targeting can no longer satisfy researchers' need for precise targeting. To achieve targeting of MCF-7 cells, Yao et al. used heavy chain ferritin (HFn) nanocages as carriers coupled to CDs and encapsulated with the chemotherapeutic drug DOX. They demonstrated that ROS generated by CDs inhibited cells by damaging DNA, and neutral single-cell gel electrophoresis showed the longest staining tails for laser-irradiated HFn (DOX)/CDs, suggesting that DNA damage was most effective with simultaneous action of CDs and DOX. Also, ferritin breakdown under acidic conditions favored DOX release, with cumulative DOX release increasing to 77.8% at pH = 5 for 72 h [23].

### In antibacterial

The use of CDs for photodynamic antimicrobials is aimed at solving the growing problem of antibiotic resistance. The antimicrobial behavior mediated by CDs as PSs can be attributed to the following aspects: (1) The abundant groups on the surface of CDs make their surfaces have different charges, and when the CDs come into contact with the bacteria through electrostatic adsorption, they will change the electrochemical potential of the bacterial membrane and thus increase the permeability of the membrane, which will cause the bacteria to die after destroying the membrane structure. Death of bacteria after destroying the membrane structure. Researches have proved that the combination of positively charged CDs and negatively charged bacterial cell membranes leads to membrane damage, which is currently recognized as the main mechanism of bacterial killing by CDs [125–127]. (2) Upon receiving light energy delivered by PDT, CDs can generate large amounts of ROS through a photoelectron transfer mechanism to destroy bacterial outer membranes/proteins and other biomolecules [128, 129]. (3) The nanoscale particle size of CDs is easily ingested by bacteria, interfering with the intracellular components of bacteria and inducing the occurrence of bacterial programmed death [130, 131]. The advantage of utilizing photodynamic antibacterials with CDs is that PDT works through irreversible oxidative damage, and therefore little or no drug resistance develops. In addition to this, CDs are effective against bacteria that are otherwise resistant or sensitive to drugs, as this damage is generally considered to be non-specific [132].

#### CDs as photosensitizers and carriers in PDT

**Fig. 9:**
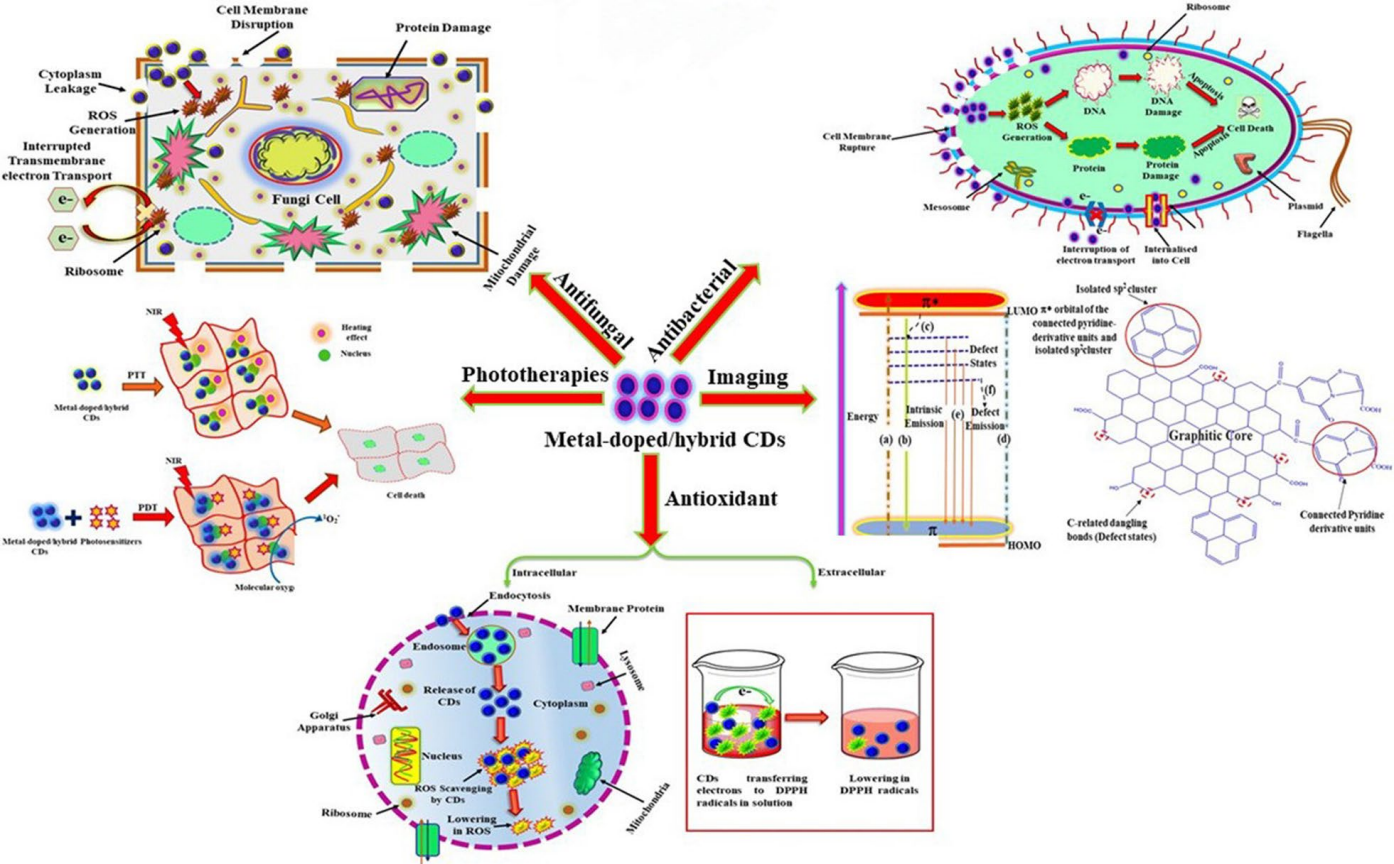
Metal-doped carbon dots for biomedical applications, especially in antimicrobials [134]. Copyright 2021, Elsevier publishing group

From the mechanism of ROS generation by PDT, a sufficient number of triplet state electrons is crucial. Generally, the spin–orbit coupling (SOC) constants between the singlet and triplet states are expanded by introducing heavy atoms such as iodine, selenium, tellurium and bromine into the CDs, which can further improve the efficiency of ROS generation from CDs effectively (Fig. 9) [133, 134]. Rachael et al. creatively doped CDs with bromine atoms to realize phosphorescence emission from fluorescent CDs. They found that the Type I and Type II photosensitization effects possessed by such carbon dots (BrCND) were due to the heavy-atom effect to obtain a triplet state character. Meanwhile, BrCND had higher photosensitivity than the CDs without bromine. Upon exposure to 365 nm UV light irradiation, BrCND showed significant inhibition of E. coli, Listeria monocytogenes and S. aureus. This inhibition was reported to cause a 2–5 log reduction in bacterial viability [135].

In order to improve the photosensitivity of CDs for photodynamic antimicrobial applications, some scholars have suggested that the fluorescence and phosphorescence quantum yield of CDs can be improved by doping with N. Wu et al. prepared a variety of N-doped CDs by using ethylenediamine as the nitrogen source, and the results showed that there was a positive correlation between the phosphorescence quantum yield of the four types of N-doped CDs and the photo-oxidative efficiency. The inhibition efficiency of the N-doped CDs against Salmonella and E. coli was higher and faster (92% and 86%) than that of the molecular PS phloxine B (40% and 55%) in PDT antimicrobial experiments [79].

Copper-based photocatalysts have been shown to have the ability to generate ROS efficiently [136, 137]. Forrest et al. coordinated copper with N to generate copper-centered functionalized N-doped carbon dots (Cu/NDC), and they found that Cu/NDC had a 1.4 ns longer decay time constant than NCD with undoped Cu. This extended lifetime facilitated the separation of photogenerated electron–hole pairs, which enhanced the performance of Cu/NCD for ROS generation. In the antimicrobial experiments, the E. coli colony counts in the Cu/NCD group subjected to light irradiation were significantly reduced, while the control group showed no significant damage. Transmission electron microscopy results also revealed the bactericidal activity of Cu/NCD. In the presence of Cu/NCD, 365 nm light exposure caused severe membrane damage and deformation of E. coli cells [138]. Several studies have demonstrated that CDs doped with atoms have abundant surface states and unique photoelectron transfer properties, making them a potential choice for optimizing the antimicrobial properties of PDT.

**Fig. 10:**
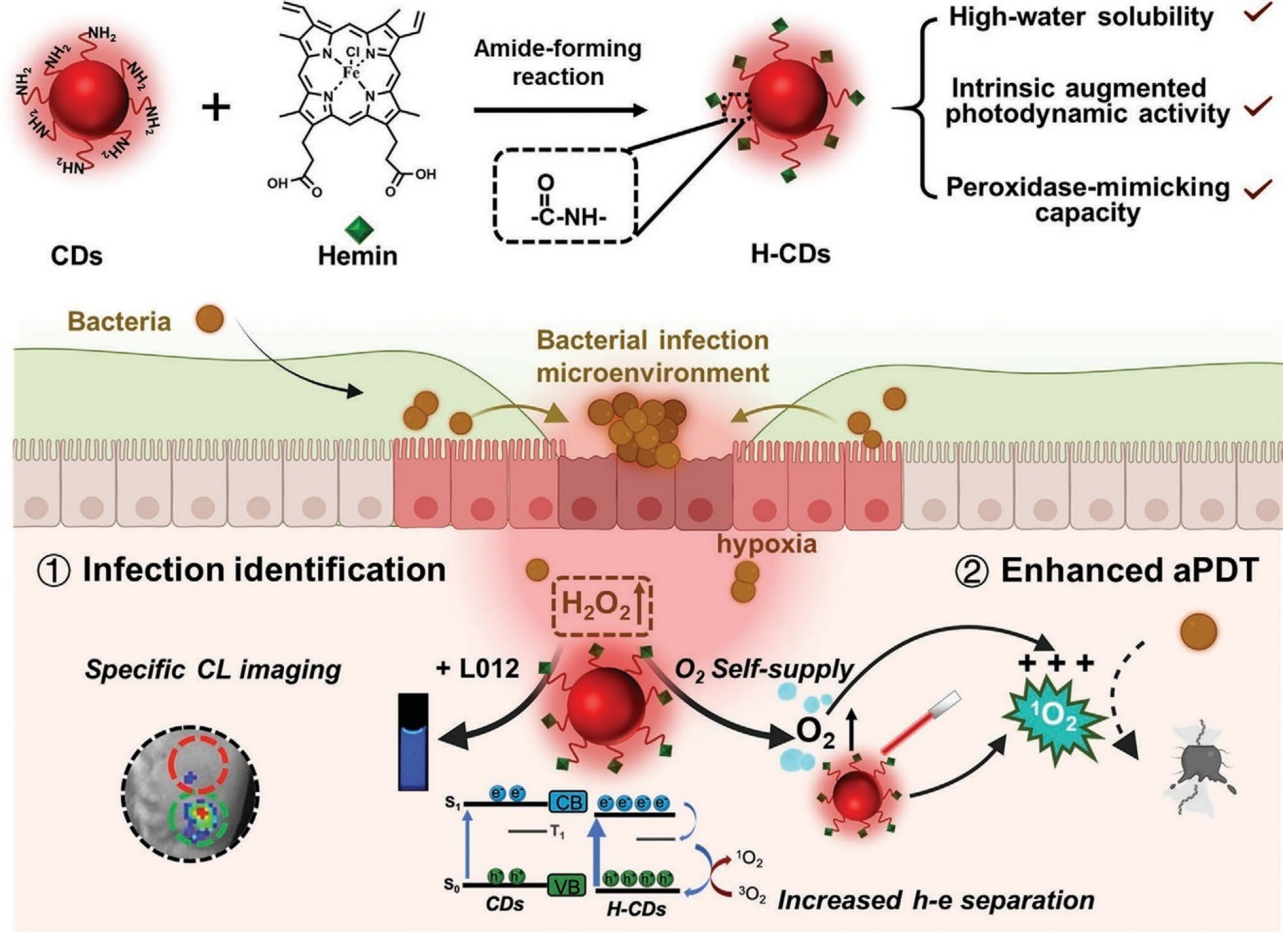
Schematic illustration of H-CD in specific imaging and enhancement of PDT for bacterial infections [139]. Copyright 2023, John Wiley and Sons publishing group

To overcome the defects of poor hydrophobicity of conventional PSs and to simultaneously achieve specific chemiluminescence for bacterial infections and enhanced antimicrobial PDT, heme chloride-modified carbon dots (H-CDs) were designed by Cheng et al. Heme chloride is important in physiological signaling pathways with catalytic activity, and its peroxidase-like nature can catalyze the generation of large amounts of oxygen to supply the oxygen-demanding PDT process. These modified H-CDs could promote photoactivated e–h separation, which significantly improved the electron–hole separation efficiency leading to more efficient killing (Fig. 10). The results showed that under 660 nm laser irradiation, H-CDs exhibited significant bactericidal effects against all types of bacteria. When the concentration of H-CDs was increased to 300 µg/mL, the antibacterial efficiency was close to 100% [139].

Nie et al. creatively used electrostatic spinning to embed carbon dots into polyacrylonitrile nanofibers (PAN-CQDs NFs), which were not only recyclable but also provided a large surface area for bacterial attachment. Meanwhile, the CDs uniformly attached within the nanofibers retained the PL characteristics and were more sensitive to PDT inactivation at a fixed illumination intensity compared to the Gram-positive bacteria S. aureus, the Gram-negative bacteria E. coli, Pseudomonas aeruginosa and Bacillus subtilis with an inactivation up to about 6 log units. This suggested that CDs inactivate both Gram-positive and negative bacteria, but were slightly weaker against clustered S. aureus [132].

In addition, the surface functionalization of CDs has a significant effect on the antimicrobial effect of PDT. Yang et al. prepared CDs with –CH_3_-terminal uncharged and –NH_2_-terminal positively charged CDs on the surface, respectively. The results showed that positively charged molecules on the surface would enhance the interaction of CDs with the bacterium Bacillus subtilis, thus promoting the light-activated antimicrobial performance of CDs [140]. Therefore, the elemental composition or surface modification of CDs can be considered to achieve enhanced photosensitization properties and thus antibacterial efficacy. Similarly, Liu et al. used dopamine hydrochloride as a passivator to modify and synthesize CyCDs, which had positively charged amino groups on their surfaces that could rapidly bind to the negatively charged phosphoric acid in the bacterial cell wall [141].

#### Combination therapy with PDT

PDT has limitations in treating bacterial infectious diseases (as mentioned below). PTT, on the other hand, uses PSs to convert photon energy into heat, which can effectively destroy bacterial tissues and prevent biofilm formation. However, PTT requires laser irradiation with reasonable power to generate heat, and its effectiveness is highly dependent on the photothermal conversion efficiency of CDs [142, 143]. The respective limitations of PDT and PTT provide inspiration for combining the two invasive treatments.

**Fig. 11:**
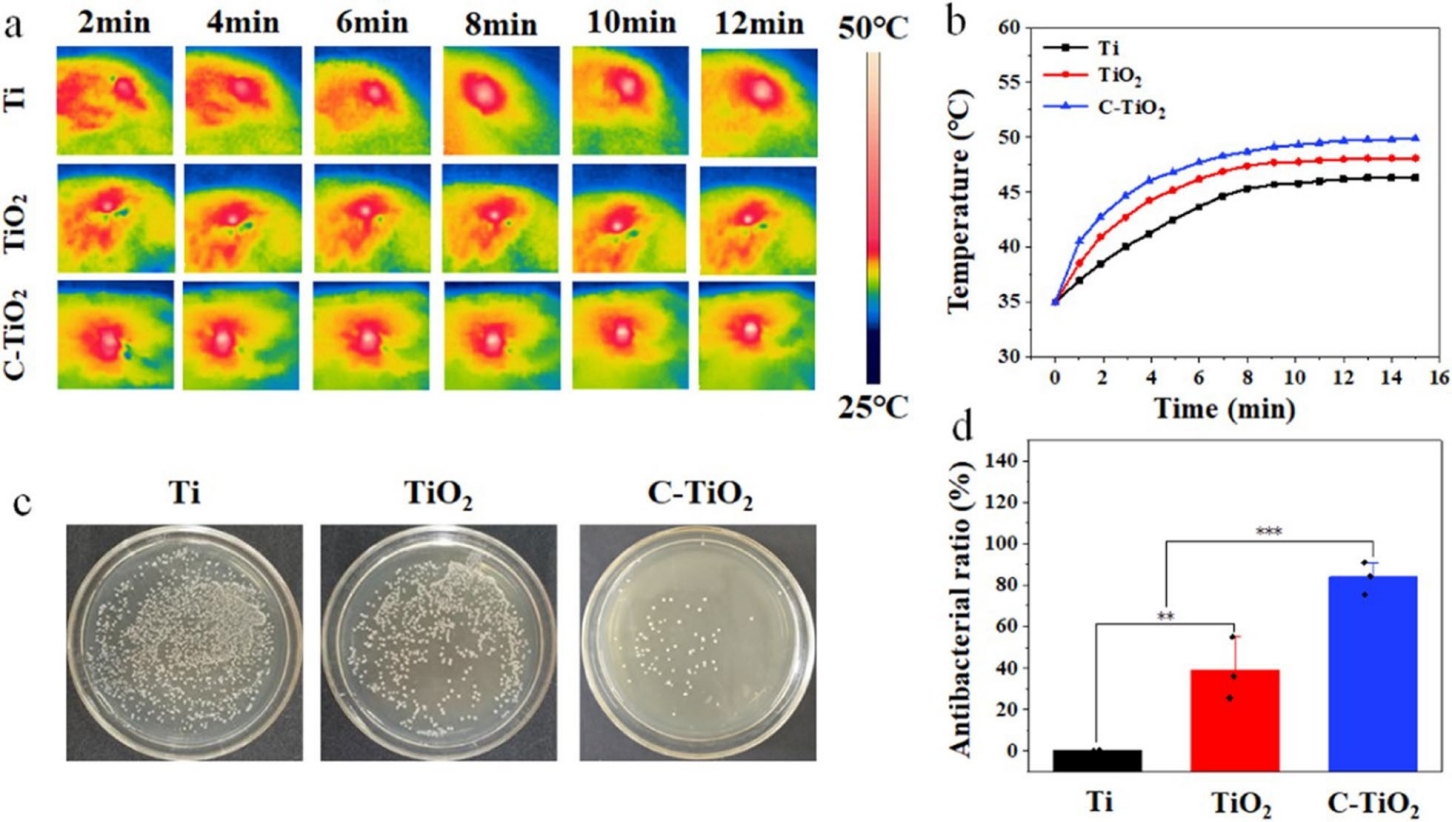
Photo thermograms of in vivo dual-light (808 nm NIR + 660 nm VL) irradiation for 15 min, temperature changes, colony counts and in vivo antimicrobial efficiency. **a** Photothermal graphics and **b** temperature variation of Ti, TiO_2_ and C-TiO_2_ NA under dual light (808 nm NIR + 660 nm VL) irradiation 15 min in vivo; **c** Pictures of S. aureus colonies; **d** Antibacterial efficiency in vivo [129]. Copyright 2022, Elsevier publishing group

Ho et al. doped CDs in TiO_2_ nanorod arrays (C-TiO_2_ NR) to achieve efficient sterilization under co-irradiation of 660 nm visible light (VL) and 808 nm NIR. In rat subcutaneous S. aureus infection models, the maximum temperature at the implantation site was found to be up to 50 °C and the antimicrobial efficiency was found to be up to 85% after 15 min of dual-light irradiation (Fig. 11). In this study, they found that the CDs could undergo interfacial charge transfer with TiO_2_ to enhance the photocatalytic performance, and the doping of CDs further improved the photothermal performance of TiO_2_ NR [129].

**Fig. 12:**
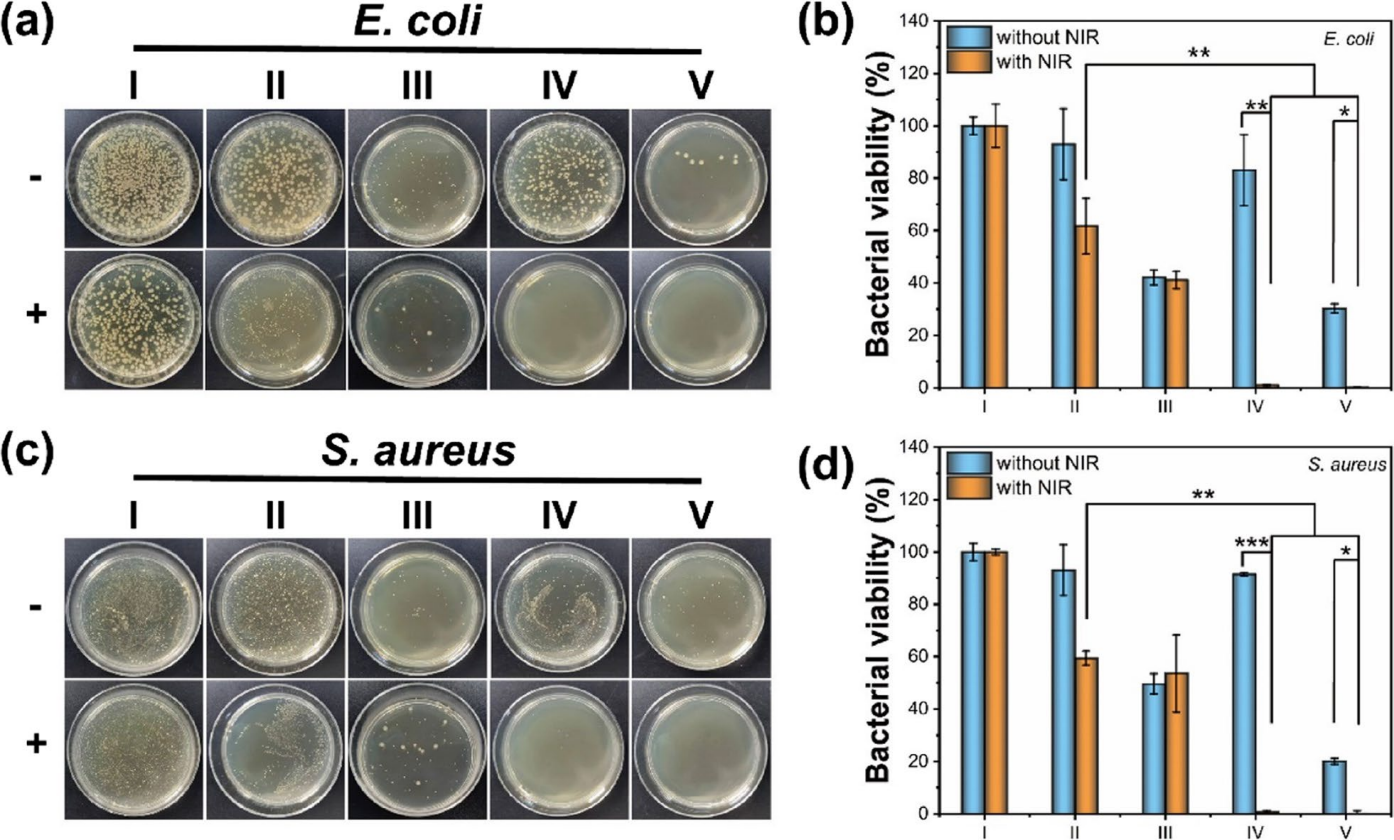
Schematic diagram of the plate for E. coli and S. aureus after different treatments and antibacterial rates of E. coli and S. aureus after different treatment methods. **a** E. coli and **c** S. aureus after different treatments (I: blank group, II: OD/HPCS/FeCDs hydrogel, III: OD/HPCS-*g*-EPL hydrogel, IV: OD/HPCS/Fe-CDs hydrogel + H_2_O_2_, V: OD/HPCS-*g*-EPL/Fe-CDs hydrogel + H_2_O_2_. Antibacterial rates of **b** E. coli and **d** S. aureus after different treatment methods [144]. Copyright 2023, American Chemical Society publishing group

CDs doped with iron can alter the internal electronic environment and exhibit higher photosensitizing activity by increasing the utilization of CDs active sites. Dong and his team utilized Fe-CDs as PSs loaded on hydrogels formed by oxidized dextran anhydride and hydroxypropyl chitosan-*graft-ε*-poly (_L_-lysine) for synergistic antimicrobial peptides-PTT-PDT for the treatment of bacterial infectious diseases. They noted that with the hydrolysis of the amide bond in the hydrogel, the positively charged *ε*-Poly(_L_-lysine) could be selectively adsorbed on the bacterial surface through electrostatic interactions with the negatively charged bacterial cell membranes, leading to their depolarization. Meanwhile, Fe-CDs generated heat and catalyzed the production of •OH under NIR laser irradiation. The result led to an antibacterial rate of about 99% or more against E. coli and S. aureus (Fig. 12). The above results indicated that the composite hydrogel-CDs had great potential for application as wound dressings for the treatment of bacterial infections, highlighting the high efficiency and safety of the combined therapy [144].

Curcumin (Cur) is a commonly used PS with bioactivities such as anti-inflammatory, antioxidant, anticancer, and antimicrobial. Cur exhibits antimicrobial effects against E. coli, Burkholderia cepacian and Vibrio parahaemolyticus under UV and blue LED irradiation, but suffers from the drawbacks of low aqueous solubility and poor photostability [145–147]. Yan et al. developed composite system with CDs as a carrier, CDs/Cur, which improved the bioavailability and ROS yield (405 nm) of Cur due to FRET action. Meanwhile, CDs/Cur could well inherit the high photothermal efficiency of CDs under external light source (808 nm), which was mainly because of the strong light absorption of CDs. The results showed that CDs/Cur could kill E. coli up to 100% of 1 M CDs/Cur concentration. In contrast, it showed a stronger lethal effect on S. aureus, with 100% mortality at the lowest concentration of 0.10 nM [148]. For PDT treatment alone, the addition of PTT caused more severe damage to the bacterial biofilm. The above provide a new reference system for the inactivation of bacteria by conventional CDs-mediated PDT, which further realizes efficient phototherapy.

## Drawbacks of CDs in PDT

However, there are still some difficulties to be overcome to truly achieve clinical applications.

### Oxygen deficient environments

PDT is an oxygen-dependent process, but the tumor hypoxic microenvironment and special anoxic environments where anaerobic bacteria survive limit the effective performance of PDT. As PDT progresses, hypoxia becomes more and more severe and leads to a series of pathophysiological changes such as angiogenesis, invasion and tumor metastasis, among many other adverse consequences [149]. There have been some researches on addressing the lack of oxygen supply to the PDT, but it is not far enough along. Therefore, it is crucial to develop different strategies to prepare oxygenated CDs to overcome hypoxia-related PDT. Type I PDT exerts its cytotoxic effects by reacting with components of the tumor microenvironment through electron transfer and generating ROS, which makes Type I PDT less dependent on O_2_ and allows it to generate large amounts of ROS under hypoxic conditions. Type I PDT even possesses the ability to generate small amounts of O_2_ in situ and alleviate hypoxia [150]. Metal oxide nanomaterials generate electron–hole pairs during photoexcitation and are in a charge-separated state that favors electron transfer, which is compatible with Type I PDT and reduces O_2_ dependence. Iron-doped CDs (Fe-CDs) can confer photo-enhanced peroxidase (POD)-like activity to CDs. It was demonstrated that Fe-CDs generated heat and ROS to kill E. coli and S. aureus (antimicrobial rates of 99.85% and 99.68%) under NIR illumination at 808 nm, 2 W/cm^2^. Moreover, it was shown that the photothermal conversion efficiency of Fe-CDs was 35.11%, which produced toxic effects on bacteria without affecting healthy tissues. The temperature of the aqueous dispersion of Fe-CDs and CDs increased to 45.5 ℃ at the same concentration, whereas the temperature change in the control group was negligible. In terms of PDT, the effect of Fe-CDs was enhanced by catalyzing the production of lethal •OH from H_2_O_2_. In addition, the treatment contributed to neovascularization, fibroblast proliferation and collagen deposition, thus exhibiting a promotive effect on infected wound healing [151].

### Targeted capacity

CDs are small in size (< 10 nm), and nanoparticles accumulate to the tumor site through prolonged solid tumor circulation time and EPR properties. EPR effect generally requires a nanoparticle size of 20–200 nm, so it is difficult for unmodified CDs to be passively targeted to tumors via EPR effect, and additional active targeting or modification is required to obtain nanoparticles with optimal tumor targeting and distribution nanoparticles with optimal tumor targeting and distribution capabilities. A better solution may be to modify the basic properties of CDs nanoparticles, such as size, shape and surface properties, to achieve long cycling ability, tumor enrichment, deep penetration and better intracellular distribution. In addition, CDs have higher photosensitizing activity after modification, which can improve the efficiency and therapeutic effect of PDT [152]. Targeting key sites associated with metabolic growth of cancer cells not only enhances the uptake of CDs but also maximizes the killing effect of ROS. Intracellular targeting of organelles, such as targeting mitochondria, lysosomes and endoplasmic reticulum, can effectively induce apoptosis or necrosis in tumor cells with low ROS requirement. It is also important for guiding the selection of surgical scope and the evaluation of postoperative therapeutic efficacy. Therefore, it is equally crucial to develop CDs with the function of targeting tumor cells [153].

### *Surface charge and *in vivo* circulation*

When nanoparticles are < 10 nm in size, they are rapidly excreted by the kidneys. This means that CDs often have difficulty in achieving a balance between circulatory capacity and permeability [35]. Positively charged nanoparticles have higher nonspecific uptake rates in most cells, while nanoparticles with neutral and negative surface charges have longer circulating half-lives due to reduced adsorption of serum proteins [154]. The improved permeability and permeability of positively charged CDs is the key to increased drug bioavailability and superior therapeutic efficacy. It is also possible to construct CDs of appropriate size and morphology to modulate the metabolism and excretion of nano-sensitizers in vivo. However, it is important to note that high zeta potentials and large particle sizes can increase cytotoxicity and impair colloidal stability [155, 156]. Therefore, efforts must be made to achieve an optimal balance of surface charge and in vivo circulation of CDs to maximize therapeutic efficacy [157].

### Toxicity in vitro and in vivo

Moreover, there are few data on the pharmacokinetics and pharmacodynamics of CDs, and the biocompatibility and long-term toxicity need to be explored. Although many studies have shown that CDs have low toxicity, lethal doses and inhibitory concentrations in vivo models are unknown detailed biosafety studies will ensure successful clinical applications. Not all CDs are synthesized by the same pathways and precursor substances and depend on a variety of factors, so independent toxicity testing experiments should be available for each unique CDs. However, most toxicity studies have been demonstrated by in vitro methods in which experimental conditions are tightly controlled, but the in vivo physiological environment presents complex and often unpredictable responses to foreign substances [158]. At the same time, the process of administering various intravenous CDs when performing in vivo studies alters physiological systems and disrupts pharmacokinetics, depending on the timeline and outcome of the injection, circulation, metabolic activity, biodistribution, renal excretion, and surface bio-interface interactions [156]. It should not be overlooked that ROS generated during PDT may also lead to normal cell death. In order to solve the above problems, it is necessary to establish controllable CDs synthesis strategies and application methods, and to conduct in-depth studies on possible toxic side effects and complications [54, 159, 160].

### Clinical translation

Various therapeutic studies of CDs are currently at the cellular and animal levels, but differences between animal models and humans need to be considered in clinical translation. The development of clinical diseases is much longer and more variable than "fast-growing" tumors and relatively homogenous infections in mice. The rate of progression, immune infiltration, level of inflammation, metabolism and signaling in animal models may affect the sensitivity of the diseases to treatment with CDs in PDT [153]. Therefore, preclinical experiments should be conducted with due consideration of these differences to obtain more reliable results.

Although CDs face numerous challenges in PDT, all of these issues will be gradually solved with the continuous development of related fields such as biology, chemistry and nanomedicine, which provide multiple opportunities to address the existing challenges.

## Conclusions and perspectives

In conclusion, CDs, as a new member of the photoluminescent carbon family of nanomaterials, have good potential for applications in PDT for disease therapy due to their unique physicochemical properties. CDs have good biocompatibility and high PDT efficiency as PSs while being classical PS carriers can solve most of the current problems, such as phototoxicity and low water dispersion of conventional PSs. The chemical structure on the surface of CDs can bind to antibodies and specific proteins to aid localization and achieve specific therapy. Meanwhile, we summarize the role of doped and modified CDs as antimicrobial PSs and propose the importance of surface functionalization to improve the photosensitizing properties of CDs.

Due to their good PL effect, CDs can not only be used as PSs or carriers in PDT of tumor and bacterial infectious diseases therapy, but also fluorescence emission provides CDs with the ability to guide therapy by bioimaging. Image-guided PDT using CDs would provide an excellent way to detect drug traces in organisms in real time, and may also enable an organic combination of diagnosis and therapy.

## Data Availability

Data sharing is not applicable to this article as no datasets were generated or analyzed during the current study.

## Abbreviations

CDs: Carbon dots
PDT: Photodynamic therapy
PSs: Photosensitizers
ROS: Reactive oxygen species
PTT: Photothermal therapy
PL: Photoluminescence
FDA: Food and Drug Administration
^1^O_2_: Singlet oxygen
5-ALA: 5-Aminolevulinic acid
MOFs: Metal organic frameworks
AFM: Atomic force microscopy
N: Nitrogen
P: Phosphorus
S: Sulfur
B: Boron
F: Fluorine
NIR: Near-infrared
LED: Light emitting diodes
SDT: Sonodynamic therapy
HA: Hyaluronic acid
DPP: Diketopyrrolopyrrole
CTS: Chitosan
MTT: 3-(4,5-Di-methylthiazol-2-yl)-2,5-diphenyltetrazolium bromide
FRET: Fluorescence resonance energy transfer
FLIM: Fluorescence lifetime imaging microscopy
Mn-Pc: Manganese phthalocyanine
HB: Hypocrella Bambusae
CHT: Chemotherapy
DOX: Currently doxorubicin
FTIR: Fourier transform infrared
EDC: 1-(3-Dimethylaminopropyl)-3-ethylcarbodiimide hydrochloride
NHS: N-Hydroxy succinimide
CA: Citric acid
EDA: Ethylenediamine
SOC: Spin–orbit coupling
EPR: Enhanced permeability and retention
